# Eerdun Wurile, a Mongolian medicine, alleviates neuronal damage and improves neurological dysfunction after cerebral hemorrhage by activating the PI3K/AKT signaling pathway

**DOI:** 10.1186/s13020-026-01459-0

**Published:** 2026-07-07

**Authors:** Wenhui Yang, Yilu Li, Xueying Wang, Yushi Tang, Zhenxing Tao, Pengpeng Li, Shiqing Du, Zhenqian Mu, Xiaojie Lu, Xudong Zhao

**Affiliations:** 1 Department of Neurosurgery, Jiangnan University Medical Center (Wuxi No. 2 People’s Hospital), Wuxi, 214002 People’s Republic of China; 2 Department of Neurosurgery, Wuxi No. 2 People’s Hospital, Affiliated Wuxi Clinical College of Nantong University, Wuxi, 214002 People’s Republic of China; 3 Pharmaceutical Department, Inner Mongolia Forestry General Hospital, Hulunbuir, 022150 People’s Republic of China; 4 Neuroscience Center, Wuxi School of Medicine, Jiangnan University, Wuxi, 214122 People’s Republic of China; 5 Second Affiliated Hospital of Soochow University, Suzhou, 215004 People’s Republic of China; 6 Department of Anesthesiology, The Second Affiliated Hospital of Chongqing Medical University, Chongqing, 400000 People’s Republic of China; 7 Department of Neurosurgery, Northwest University Affiliated 1s, Hospital, Xi’an, 710000 People’s Republic of China; 8 Yangxin County People’s Hospital, People’s Republic of China Huangshi,

**Keywords:** Intracerebral hemorrhage, Eerdun Wurile, Neuron, Apoptosis, Inflammation

## Abstract

**Background:**

This study aims to evaluate the neuroprotective effects of the Mongolian medicine Eerdun Wurile (EW) against intracerebral hemorrhage (ICH) and explore its underlying mechanisms. ICH, a severe subtype of stroke, is characterized by a complex pathological process, with no specific treatments currently available. EW, a traditional Mongolian medicinal formula, has a long history of use in treating central nervous system disorders, but its mechanism of action in ICH remains poorly understood.

**Methods:**

A mouse model of ICH was induced through stereotactic intracerebral injection of Collagenase Type IV. Behavioral assessments, histological examinations, and RNA sequencing were conducted to assess the effects of EW and elucidate its mechanism of action in mitigating neuronal injury post-cerebral hemorrhage.

**Results:**

EW administration improved a range of ICH-induced deficits, including neurological and neurofunctional impairments, anxiety-like behaviors, and cognitive dysfunction. EW protected neurons from injury and apoptosis in both in vitro and in vivo models. Additionally, EW treatment suppressed the activation of astrocytes and microglia, reducing neuroinflammation, preserving blood–brain barrier integrity, and alleviating synaptic damage. RNA-seq analysis revealed that the PI3K/AKT signaling pathway plays a key role in EW's neuroprotective effects. EW activates this pathway, modulating the balance of Bcl-2 family proteins, thereby reducing neuronal apoptosis.

**Conclusion:**

EW confers neuroprotection through multiple mechanisms. These findings provide strong experimental evidence supporting the potential clinical application of EW as a therapeutic agent for ICH.

**Supplementary Information:**

The online version contains supplementary material available at 10.1186/s13020-026-01459-0.

## Introduction

Intracerebral hemorrhage (ICH) is characterized by non-traumatic bleeding within the brain tissue, constituting approximately 10% to 15% of all stroke cases [1, 2]. Epidemiological studies show that the annual incidence of ICH ranges from 0.01% to 0.03% per 100,000 individuals, with a 30-day mortality rate as high as 50% [3]. The primary pathophysiological mechanism involves the rupture of small cerebral blood vessels or microaneurysms, often resulting from chronic hypertension, with sudden blood pressure spikes frequently acting as triggers [4]. Following ICH, the hematoma increases intracranial pressure and impedes blood flow to adjacent brain regions, leading to ischemia and neuronal death [5]. Moreover, ICH initiates a secondary injury cascade marked by severe neuroinflammation, oxidative stress, and blood–brain barrier (BBB) disruption [6–8]. This neuroinflammatory response includes the rapid activation of resident glial cells (microglia and astrocytes), followed by the recruitment of peripheral leukocytes to the perihematomal region [9].

Current therapeutic strategies for ICH primarily aim to limit hematoma expansion and prevent complications. Despite extensive research into ICH treatments, no specific therapy has yet been developed to address brain injury caused by ICH [10–12]. However, several traditional Chinese medicine (TCM) formulations have shown promising therapeutic effects in both animal models and clinical trials, demonstrating potential in alleviating damage from brain hemorrhage [13, 14].

Eerdun Wurile (EW), a traditional Mongolian remedy, has a long history of use in treating central nervous system and cardiovascular disorders. This herbal formulation contains 29 components, including Terminalia chebula Retz (fruits), Carthamus tinctorius (flowers), and Gardenia jasminoides Ellis (fruits), and is indicated for nerve injury, ICH, and cerebral thrombosis. Mechanistically, EW promotes a shift in microglial polarization from the pro-inflammatory M1 phenotype to the anti-inflammatory M2 phenotype, highlighting its neuroprotective and anti-inflammatory effects. This shift reduces neuroinflammation by suppressing pro-inflammatory cytokines and enhancing anti-inflammatory mediators, thereby mitigating neuronal apoptosis [15].

As demonstrated by previous research [16], the beneficial effects of EW on postoperative cognitive dysfunction are mediated through its suppression of the TLR4/NF-κB pathway. However, the neuroprotective and potential neuroregenerative mechanisms of EW in the context of ICH remain poorly understood. This study first confirms the improvement of motor function in ICH mice treated with EW through behavioral assessments. Subsequently, the mechanism of action of EW was explored using RNA sequencing (RNA-seq) in an in vitro model of simulated ICH. KEGG analysis revealed the PI3K/AKT signaling pathway as a significantly enriched hit, highlighting its critical role in regulating cell survival, proliferation, apoptosis, and inflammation, suggesting its potential involvement in neuroprotection [17]. Activation of the PI3K/Akt pathway after ICH protects neurons from inflammatory mediators and oxidative stress, thereby reducing neuronal apoptosis [18, 19]. By promoting neuronal survival and repair, the PI3K/Akt pathway is essential for the observed reductions in brain tissue damage and improvements in neurological function following ICH [20]. AKT, a key component of the PI3K/AKT signaling cascade, regulates various anti-apoptotic proteins, including members of the Bcl-2 family, thereby promoting neuronal survival [21].

In summary, although EW has a long history of clinical use, the lack of detailed mechanistic research limits its broader clinical application. By integrating RNA-seq data with in vitro and animal ICH models, this study employs a multi-platform approach to elucidate the neuroprotective mechanism of EW. These findings provide strong support for its clinical use and offer a foundation for novel treatment strategies. Additionally, this work contributes to the understanding of the mechanisms underlying ethnic medicines and provides a reference for further research in this field.

## Materials and methods

The EW used in this study is a commercially available standard Mongolian medicinal pill produced by Ulanhot China-Mongolia Pharmaceutical Co., Ltd. It is registered under the National Drug Approval Code Z15021615, with the experimental batch number 230655 and a production date of June 30, 2023. This product is a legally recognized finished drug, with its formulation, medicinal parts, ingredient proportions, and manufacturing process strictly adhering to the Drug Specifications of the Ministry of Health of the People's Republic of China-Mongolian Medicine Volume (1998 edition, standard number ZZ-8358) and the National Drug Standard Supplementary Approval Document (approval number: 2018B025). The entire production process complies with the Good Manufacturing Practice for Drugs (GMP), and the quality of the raw medicinal materials meets the relevant standards of the Pharmacopoeia of the People's Republic of China (2020 edition).

EW consists of 29 Mongolian medicinal ingredients. The Latin binomial names, medicinal parts, and formulation proportions of each ingredient are provided in Supplementary Table 1. The manufacturer conducts standardized quality testing for each batch, including morphological identification, microscopic identification, thin-layer chromatography, moisture determination, weight variation, disintegration time, and microbial limit testing. All test parameters for the batch used in this study met legal standards, confirming stable and controllable quality. EW was administered by oral gavage at low (10.5 mg/10 g) and high doses (21 mg/10 g), with the dosage determined based on clinical equivalent dose conversion (body surface area conversion with the Km factor method), Mongolian medicine clinical administration guidelines, and relevant previous pharmacological studies [22, 23].

### ICH model and experimental grouping

Mice were anesthetized with 3–4% inhaled isoflurane (Qingdao Orbiepharm Co., Ltd., China) for 2–3 min until fully anesthetized. Collagenase Type IV (C5138; Sigma) was injected stereotactically into the striatum at coordinates 0.8 mm anterior to the bregma, 2.0 mm lateral to the midline, and 3.5 mm deep into the brain parenchyma [24], with a concentration of 0.07 U in phosphate-buffered saline (PBS) and an injection rate of 0.1 μl/min.

#### Experiment 1

Male C57BL/6J mice were randomly assigned to 5 groups using a random number table: Sham group, ICH group, ICH + EW-L group [25] (10.5 mg/10 g, oral gavage, starting 2 h after surgery and continuing until day 7 post-surgery), ICH + EW-H group (21 mg/10 g, oral gavage, starting 2 h after surgery and continuing until day 7 post-surgery), and the Edaravone injection (EDA) group (Boda Pharmaceutical Co., Ltd., China), which received EDA injection 2 h after surgery, continuing until day 7 post-surgery.

#### Experiment 2

C57BL/6 J mice were divided into 4 groups: Sham group, ICH group, ICH + EW group, and ICH + EW + LY294002 group, assigned using a random number table. The LY294002 group received the PI3K inhibitor LY294002 (5 nmol/μL; MedChemExpress, HY-10108, USA; dissolved in DMSO, Sigma-Aldrich, Germany) via injection 30 min prior to ICH induction [26].

All animal procedures were conducted in accordance with NIH Guidelines and approved by the Animal Care and Use Committee of Jiangnan University (Approval No.: JN. No20241230c0840530[702]).

### Behavioral studies

#### Morris water maze

Spatial learning and memory were assessed using the Morris water maze (MWM) between days 21 and 25 post-ICH induction [27]. Following a one-week adaptation period, mice underwent five consecutive days of place navigation training in the MWM. On the sixth day, a probe trial was performed with the platform removed. The ANY-maze behavioral tracking system (Stoelting) was employed to quantify spatial navigation, platform crossings, and the duration spent in the target quadrant during the 60-s trial (n = 8 per group).

#### Elevated plus maze

Anxiety-like behavior was evaluated using the Elevated Plus Maze (EPM), which consists of two open (50 × 10 cm) and two closed (50 × 10 cm, 30-cm high walls) arms. After placing a mouse in the center facing an open arm, its 5-min exploration was recorded using ANY-maze software (Stoelting). The time spent in each arm type was quantified (n = 8 per group).

#### mNSS

Neurological function was assessed using the modified Neurological Severity Score (mNSS) at three time points post-ICH: 24 h, 72 h, and 30 days. All assessments were conducted by two trained investigators who were blinded to group allocation [28–30]. The mNSS scale ranges from 0 to 18, assessing parameters such as body symmetry, gait, climbing ability, circling behavior, forelimb symmetry, compulsory circling, and whisker response. Higher scores correspond to more severe neurological impairment (n = 8 per group).

#### Brain hematoma volume

For histological analysis, mice were deeply anesthetized before transcardial perfusion with paraformaldehyde. Brains were immediately collected and serially sectioned. Hematoma volume was calculated by summing the lesion areas of all sections and multiplying the total by the thickness of individual sections [31] (n = 4 per group).

### Cell culture and treatment

#### HT22 Cells

The HT22 cell line is an immortalized mouse hippocampal neuronal line devoid of glial properties. It is extensively employed in neuroscience to investigate neuronal injury, oxidative stress, and neuroprotection in vitro, particularly in models of intracerebral hemorrhage and Alzheimer's disease, due to its responsiveness to neurotoxic stimuli and consistent culture conditions [32]. HT22 cells were cultured in high-glucose DMEM medium supplemented with 10% FBS. To simulate secondary injury after ICH, which involves the release of hemoglobin from lysed red blood cells, an in vitro system was established by exposing the cells to Hemin. This treatment replicates the brain cell injury observed post-ICH. HT22 cells were exposed to Hemin for 24 h. All in vitro experiments were performed in triplicate.

#### Primary neuron cell culture

Primary neurons were cultured as previously described [33, 34]. After coating with Poly-D-Lysine and washing with PBS, fetal mouse cerebral cortex tissue was extracted. The meninges were carefully removed under a microscope, and 4 mL of DMEM and 4 mL of trypsin were added for digestion. The digestion process was stopped by adding an equal volume of 20% FBS. After 2 min of standing, the mixture was filtered through a 40 μm cell strainer, and the resulting filtrate was plated onto culture plates. After 4 h of incubation, the medium was replaced with neuron culture medium, and the cells were maintained for 10 days before experimentation.

#### BV2 cells

BV2 mouse microglial cells were cultured in high-glucose DMEM supplemented with 10% FBS. To induce inflammation, cells were seeded in six-well plates (10^5^ cells/mL) and stimulated with 1 μg/mL lipopolysaccharide (LPS). After the LPS challenge, cells were incubated for 24 h with either PBS or EW.

#### Cell viability assay

Primary neurons, plated in 96-well plates (2,000 cells/well), were treated with EW at serial concentrations (0, 5, 10, 20, 40, 80, 160 μg/mL) following cell adhesion for subsequent experiments [35].

#### Live/dead cell staining

After treatment, neurons were washed with PBS and stained using the Calcein AM/PI Live/Dead Cell Double Staining Kit (K2247, ApexBio, USA), according to the manufacturer's protocol. Cell viability was assessed under a fluorescence microscope (ZEISS, Germany).

### Western blotting

For protein analysis, hematoma-surrounding brain tissue from mice was homogenized, centrifuged, and the supernatant collected. Following protein separation by SDS-PAGE and electrophoretic transfer onto a PVDF membrane, the membrane was incubated overnight at 4 °C with primary antibodies specific to MAP2 (Proteintech; 17,490–1-AP), GFAP (Proteintech; 60,190–1-Ig), Iba-1 (Proteintech; 10,904–1-AP), iNOS (Proteintech; 22,226–1-AP), β-actin (ABclonal; AC026), phosphor-PI3K (CST; 4228), PI3K (CST; 4292), AKT (CST; 4691 T), and phospho-AKT (CST; 4060 T). After a 1-h blocking period, the membrane was incubated with secondary antibodies, washed, developed, and the target bands were analyzed using ImageJ software.

### Immunofluorescence

Fixed frozen brain sections were stained as follows: Three days after ICH, mice were anesthetized and perfused with 0.9% NaCl, followed by 4% paraformaldehyde. Brains were post-fixed in 4% paraformaldehyde for 48 h, immersed in 30% sucrose at 4 °C, and sectioned into frozen slices. For immunostaining, sections were blocked with 5% BSA + 0.3% Triton X-100 at room temperature for 30 min, then incubated overnight at 4 °C with primary antibodies (GFAP: Invitrogen 13–0300, USA; Iba-1: WAKO 019–19741, Japan; MAP2: Proteintech 17,490–1-AP, China). After incubation with secondary antibodies for 1 h at room temperature, nuclear staining was performed using DAPI (Invitrogen YD3918381, USA). Images were captured using a confocal microscope (ZEISS, Germany).

### Transmission electron microscopy (TEM)

Synaptic remodeling following ICH was evaluated by transmission electron microscopy. Tissue samples were processed according to a standard protocol, which included fixation with 2.5% glutaraldehyde at 4 °C, post-fixation with 1% osmium tetroxide, and dehydration through a graded series of ethanol and acetone.

### Enzyme-linked immunosorbent assay

Cytokine levels (including IL-6, IL-10, TNF-a; Invitrogen, USA) were measured via enzyme-linked immunosorbent assay (ELISA) [36] after a 24-h treatment with LPS and EW.

### Serum biochemical parameters

After behavioral assessments, blood samples were collected from three mice per group. Plasma was obtained by centrifugation at 1000 rpm and 4 °C for 15 min and stored at − 80 °C. Serum levels of AST, ALP, and BUN were measured using an automatic biochemical analyzer.

### Total RNA extraction, RNA sequencing analysis, and differentially expressed gene analysis

Total RNA was extracted from the samples using TRIzol reagent, following the manufacturer's instructions. RNA quantity and purity were assessed using a Thermo Fisher Qubit 3.0 (Q33216) and an Agilent 5300 Fragment Analyzer (M5311AA). Sequencing libraries were prepared from high-quality RNA samples, all exhibiting RNA Integrity Number (RIN) values above 7.0. After RNA extraction, mRNA was isolated from 2 μg of RNA through two rounds of selection with mRNA Capture Beads 2.0 (Yeasen, 12629ES, China). Differential expression analysis was performed using DESeq2 for inter-group comparisons and edgeR for intra-sample assessments [37]. Differentially expressed genes (DEGs) (FDR < 0.05, |fold change|≥ 2) were subjected to functional enrichment analysis using the Gene Ontology (GO) and Kyoto Encyclopedia of Genes and Genomes (KEGG) pathway databases [38].

### Statistical analysis

Data are based on a minimum of three replicates per group and are presented as mean ± SEM. Statistical analysis was performed using one-way ANOVA followed by Tukey's test (GraphPad Prism 8.0), with significance indicated as ns: not significant (*p* > 0.05), *p < 0.05, **p < 0.01, and ***p < 0.001*.*

## Results

### EW alleviates neurological impairment induced by ICH

**Fig. 1:**
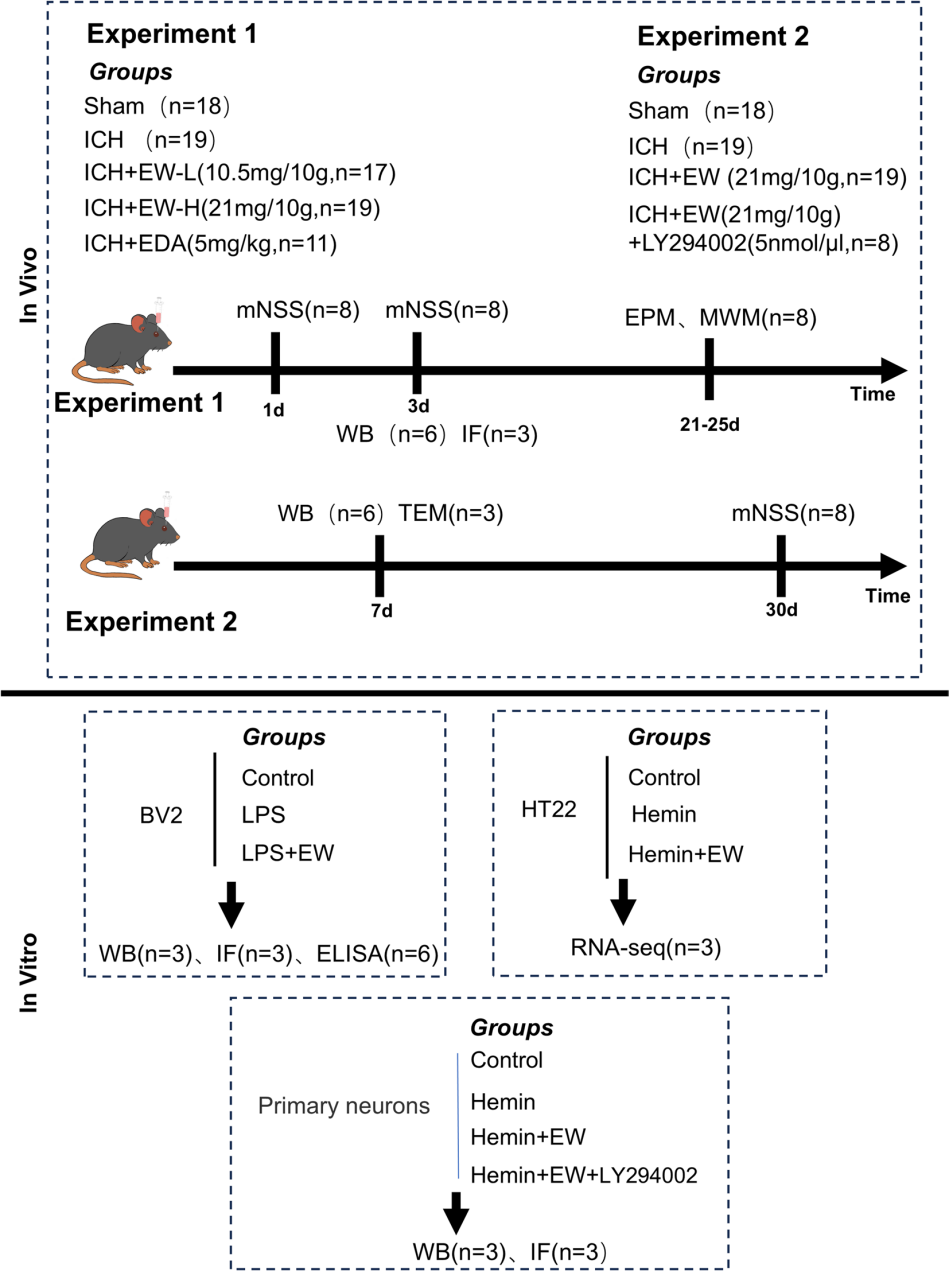
Schematic of the experimental timeline

Detailed information regarding the identification of EW is provided in Supplementary Material 1. The experimental grouping and specific design workflow are illustrated in Fig. 1, which clearly outlines the treatment regimen for each group and the experimental timeline. Compared to the Sham group, no significant changes were observed in serum ALT, AST, or BUN levels in ICH model mice. Additionally, EW treatment did not produce statistically significant differences in these indicators (Supplementary Figure S5), suggesting that EW at the experimental doses had no adverse effects on hepatic or renal function.

**Fig. 2:**
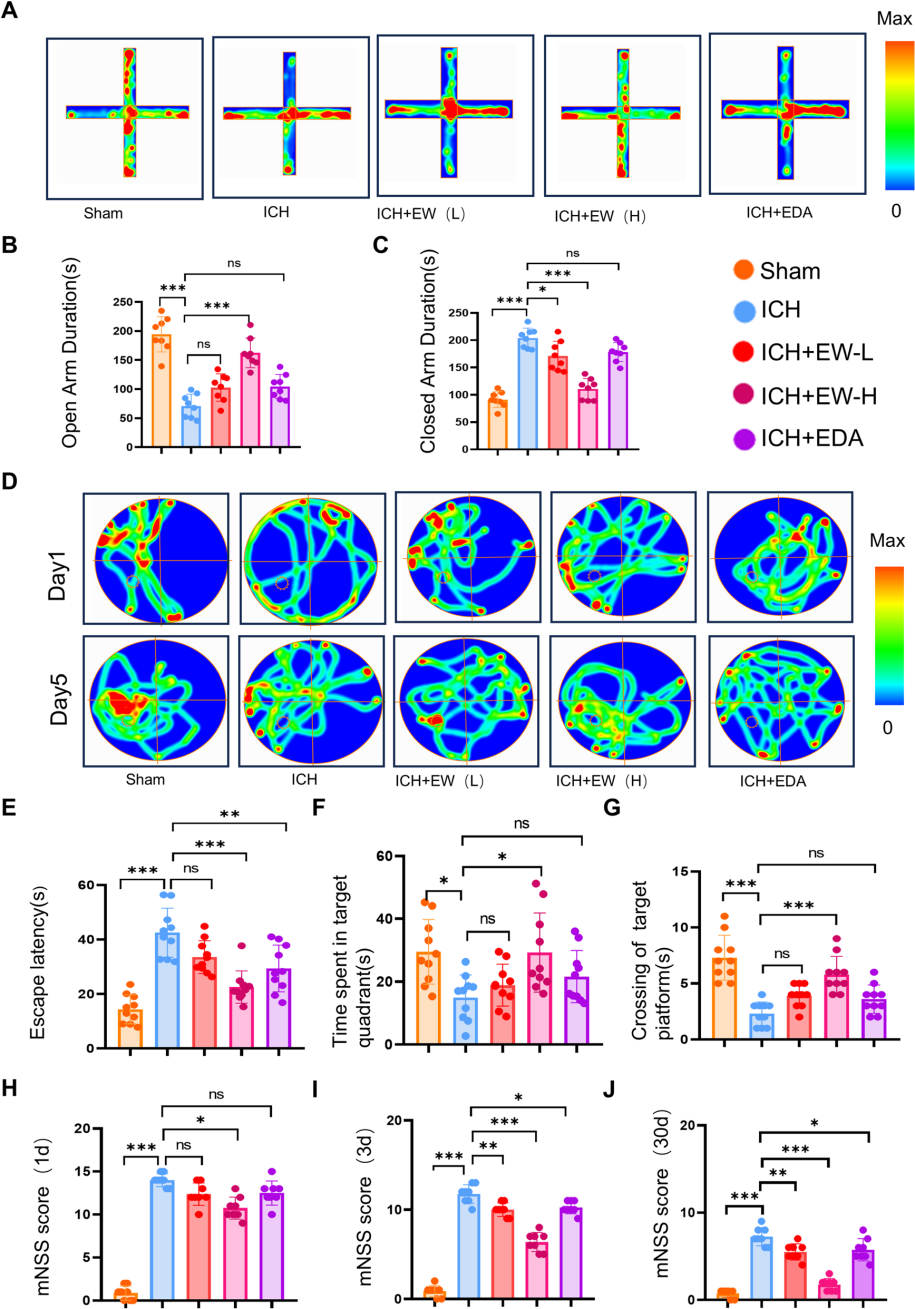
EW treatment mitigates neurological deficits induced by ICH. **A**–**C** Representative heatmaps and quantitative analysis of EPM performance. **D**–**G** MWM navigation trajectories and corresponding latency/occupancy quantification. **H**–**J** mNSS assessing sensorimotor function in ICH mice. *p < 0.05, **p < 0.01, ***p < 0.001

Behavioral tests using the EPM, mNSS, and MWM (Fig. 2) were employed to comprehensively assess the effects of EW on neurological function in ICH model mice, including evaluations of motor skills and anxiety-like behavior. The EPM was used to evaluate anxiety-like behavior, with the corresponding activity heatmap shown in Fig. 2A. Mice in the ICH group entered the open arm less frequently and spent significantly less time there than those in the Sham group (*p* < 0.001). In contrast, the EW-H group showed a marked increase in both the number of open-arm entries and the time spent in the open arms (*p* < 0.001; Fig. 2B). Although the number of closed-arm entries increased, the duration per entry was significantly reduced (*p* < 0.001; Fig. 2C). This pattern—preferencing enclosed spaces but making brief visits—indicates that acute ICH induces anxiety-like behavior with diminished exploration, which EW treatment can reverse.

Figure 2D shows the testing trajectory heatmap from the MWM experiment. The ICH group displayed a significantly longer escape latency compared to the Sham group (*p* < 0.001). In comparison, ICH mice treated with EW-H showed a significantly shorter escape latency (*p* < 0.001). Moreover, the EW-H group had a significantly shorter escape latency compared to the EDA group (*p* < 0.01; Fig. 2E). During the spatial exploration phase of the MWM, the ICH group crossed the platform significantly fewer times than the Sham group. Conversely, the EW-H group exhibited significantly more platform crossings and spent substantially more time in the target quadrant than the ICH group. Additionally, mice in the EW-H group spent significantly more time in the target quadrant than those in the EW-L and EDA groups (Fig. 2F, G; *p* < 0.001). These results suggest that cerebral hemorrhage induces learning and memory deficits, which EW treatment can ameliorate, improving spatial learning and memory function.

In this study, the mNSS was used to assess neurological impairment in a mouse model of ICH. Short-term behavioral testing revealed that the high-dose EW group significantly ameliorated neurological dysfunction in mice on both day 1 and day 3 after ICH induction. In contrast, the low-dose EW group and the EDA group showed significant improvement only on day 3 post-modeling, with statistically significant differences (*p* < 0.01) (Fig. 2H, I). These results indicate that the high-dose EW group exhibited superior improvement in mNSS scores on day 3 compared to the low-dose EW and EDA groups, leading to the selection of the high-dose EW group for further mechanistic studies. To assess the long-term effects of EW on neurological recovery, the mNSS testing period was extended to 30 days in subsequent experiments, providing a more comprehensive evaluation (Fig. 2J). The results confirmed that EW alleviated neurological impairment following ICH.

### EW has neuroprotective properties in both in vitro and in vivo studies

**Fig. 3:**
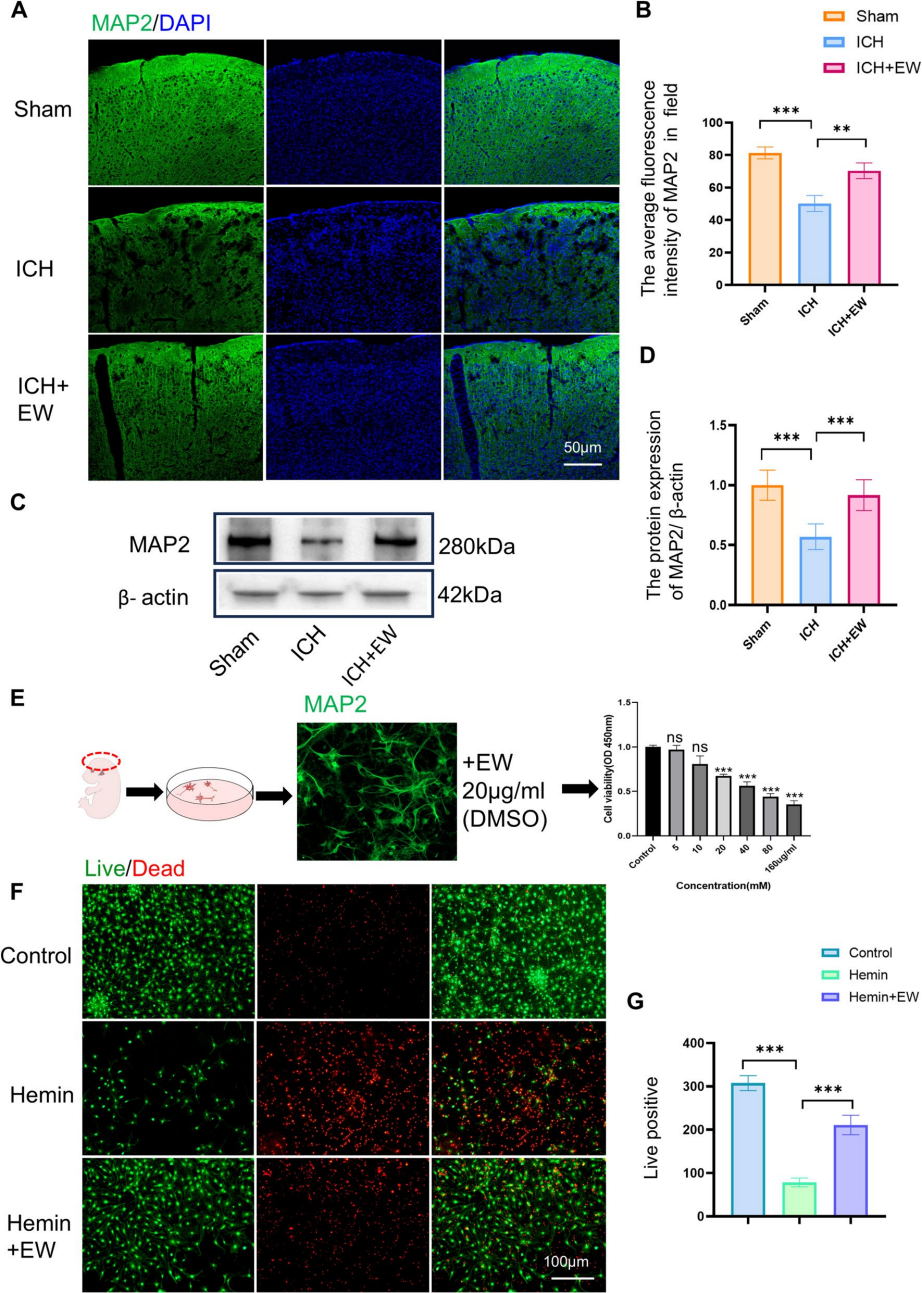
EW ameliorates neuronal damage and suppresses neuroinflammation post-ICH. **A** Schematic of the experimental timeline and imaging region, with representative coronal sections immunostained for MAP2 (green, neurons) and DAPI (blue, nuclei). Scale bar: 50 μm. **B** Quantitative analysis of MAP2-positive area in the cerebral cortex. **C**, **D** Western blot detection and quantification of MAP2 expression in brain tissues. **E** Schematic of primary neuron isolation and CCK-8 assay for EW concentration screening. **F, G** Viability of primary neurons assessed by Calcein-AM/PI staining (live: green; dead: red) and corresponding quantification. Scale bar: 100 μm. *p < 0.05, **p < 0.01, ***p < 0.001

To explore the mechanisms underlying the superior functional recovery achieved by EW treatment, the study examined the morphological characteristics and the number of neurons in the cortex. Figure 3A presents the immunofluorescence staining results for the neuronal marker MAP2. Neurons in the Sham group displayed clear and intact morphology with evenly distributed fluorescent signals. In contrast, ICH group neurons exhibited irregular fluorescence distribution and abnormal morphology in the lesion area. Following EW treatment, the fluorescence distribution became more uniform, demonstrating the neuroprotective effects of EW against ICH-induced neuronal damage. Western blot analysis further confirmed this observation, showing that MAP2 protein expression was significantly upregulated after EW treatment (Fig. 3C), providing robust evidence that EW alleviates neuronal injury.

To establish an in vitro model to assess EW's neuroprotective effects, primary cortical neurons were isolated and cultured. A concentration gradient experiment using a CCK-8 assay identified 20 μg/ml as the optimal EW concentration for subsequent experiments (Fig. 3E). Primary neurons were exposed to 10 μM hemoglobin for 24 h to induce cell damage [39, 40], followed by EW treatment for 24 h. Live/dead cell staining revealed significantly fewer dead cells in the EW-treated group compared to the ICH group (Fig. 3F). In summary, EW exhibited protective effects against neurotoxicity in both in vitro and in vivo models.

### EW inhibits astrocyte activation

**Fig. 4:**
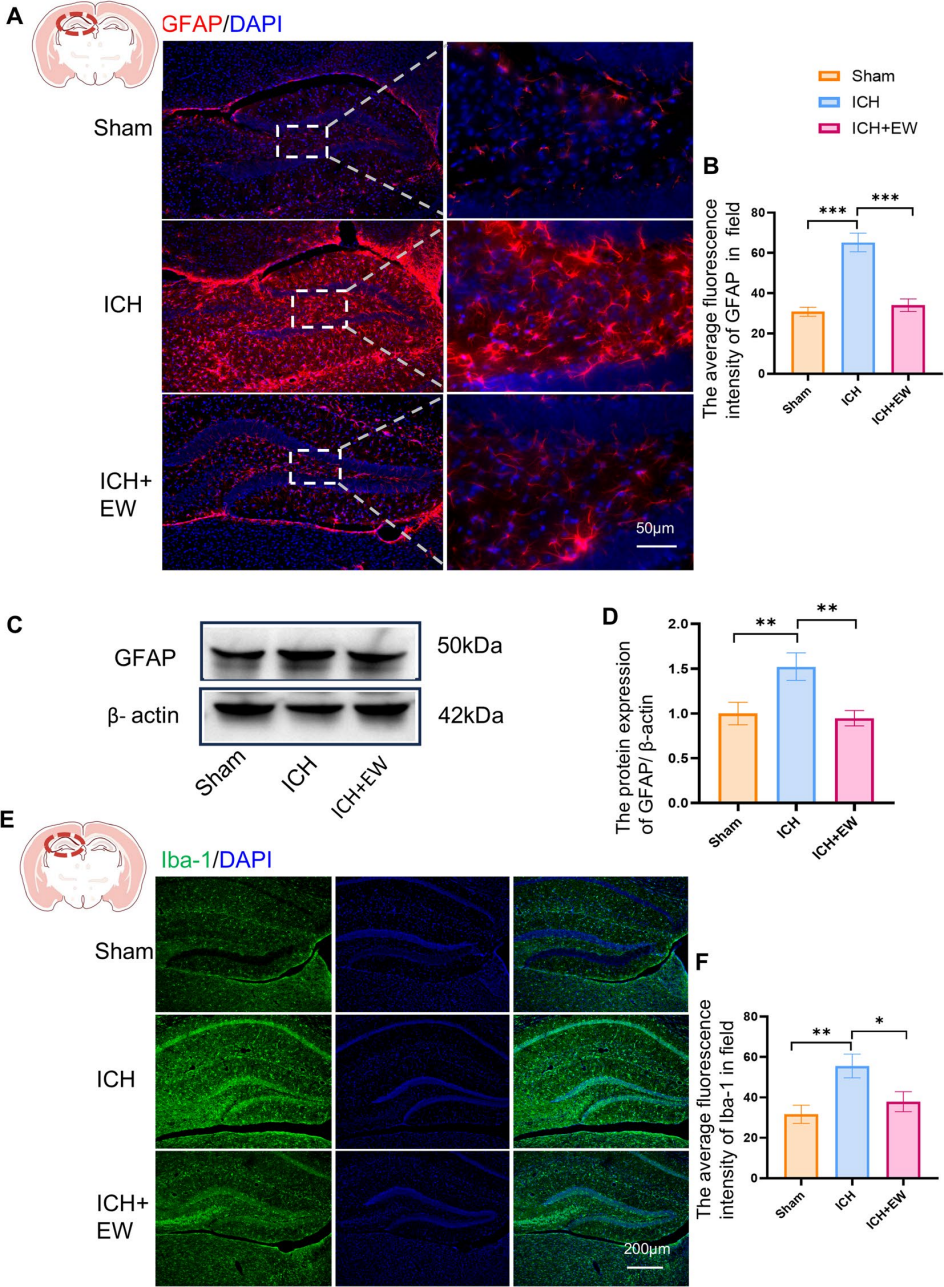
EW modulates glial activation after ICH. **A** Representative immunofluorescence images of GFAP (red, astrocytes) in the hippocampus. Scale bar: 50 μm. **B** Quantification of GFAP fluorescence intensity. **C**, **D** Western blot analysis and corresponding quantification of GFAP expression in brain tissues. **E** Representative immunofluorescence images of Iba-1 (green) in hippocampal regions. Scale bar: 100 μm. **F** Quantification of Iba-1 fluorescence intensity. *p < 0.05, **p < 0.01, ***p < 0.001

The hippocampus is a region of the brain where astrocytes are densely concentrated. Glial fibrillary acidic protein (GFAP) is a specific marker of astrocyte activation. Immunofluorescence staining revealed that GFAP fluorescence signals were significantly more concentrated in GFAP-positive (GFAP +) astrocytes in the hippocampus of the ICH group compared to the Sham group. Following EW treatment, the distribution of GFAP + astrocyte fluorescence was notably more dispersed in the ICH group (Fig. 4A, B). Quantitative analysis showed a significant increase in the number of astrocytes in the ICH group relative to the Sham group, which was markedly reduced by EW treatment. These results suggest that EW may inhibit astrocyte activation. Western blot analysis confirmed these immunofluorescence results, further supporting the conclusion that EW reduces astrocyte activation in the hippocampus (Fig. 4C, D).

### EW reduces microglial activation and neuroinflammation

Hematomas and their degradation products can trigger microglia-mediated neuroinflammation, which plays a key role in secondary injury mechanisms leading to neurological dysfunction after ICH [41, 42]. Initially, activated M1 microglia drive the inflammatory response by secreting pro-inflammatory cytokines, which contribute to extracellular matrix degradation, disruption of cell integrity, and damage to the BBB [43]. Consequently, inhibiting the activation of M1-type pro-inflammatory microglia has become a key strategy for promoting nerve repair [44].

**Fig. 5:**
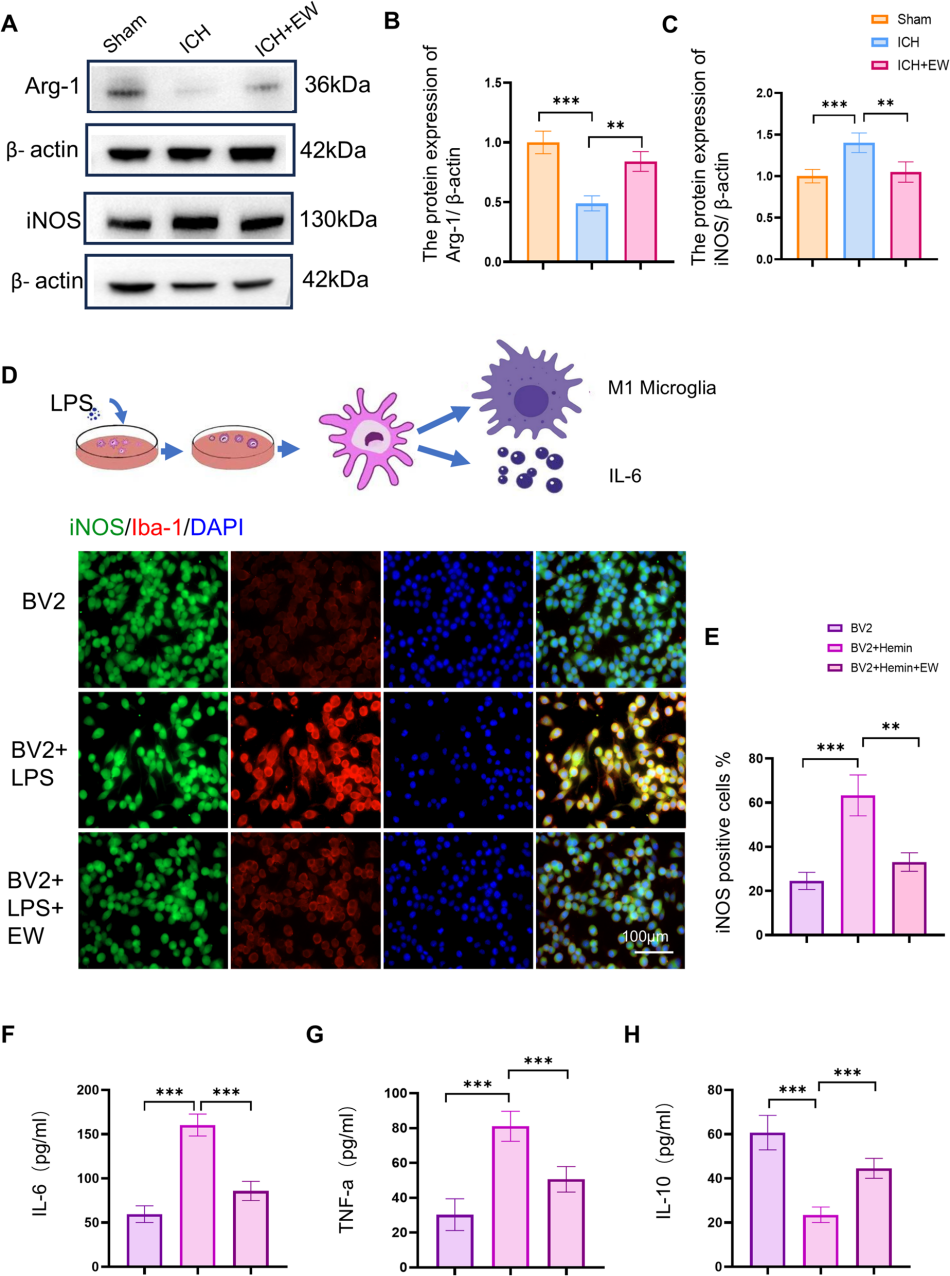
EW attenuates neuroinflammation. **A** Representative Western blot images of iNOS and Arg-1 in mouse hippocampal tissue. **B**, **C** Western blot analysis and quantification of iNOS and Arg-1 expression. **D** Schematic of the LPS-stimulated BV2 microglial assay and representative immunofluorescence images of BV2 cells labeled with Iba-1 (green, microglia marker), iNOS (red, pro-inflammatory marker), and DAPI (blue, nuclei). Scale bar: 100 μm. **E** Statistical analysis of immunofluorescence for iNOS‑positive cells. **F**–**H** IL-6, IL-10, and TNF-α levels in BV2 culture supernatants, determined by ELISA. *p < 0.05, **p < 0.01, ***p < 0.001

Microglia are highly sensitive to changes in the extracellular environment, with their resting state easily altered in response to external stimuli. To explore the regulatory effects of EW on microglial activation, mice were sacrificed after three consecutive days of treatment, and brain tissues were collected. Immunofluorescence staining was performed to assess Iba-1 expression as an indicator of microglial activation (Fig. 4E, F). Western blot analysis was also conducted to evaluate the pro-inflammatory marker iNOS and the anti-inflammatory marker Arg-1 (Fig. A–C). The results demonstrated that EW effectively modulated aberrant microglial activation, reducing neuroinflammation. To simulate the neuroinflammatory microenvironment post-ICH, cultured microglia were stimulated with LPS and treated with EW. Immunofluorescence staining was performed to examine Iba-1 expression and the pro-inflammatory marker iNOS. Results indicated that EW treatment effectively inhibited microglial polarization toward the pro-inflammatory phenotype. Specifically, after 24 h of 1 μg/mL LPS stimulation, the fluorescence intensity of iNOS in microglia was significantly lower in the EW treatment group compared to both the Sham and LPS groups, with statistically significant differences (Fig. 5A–E). To further investigate the molecular mechanism behind EW's anti-neuroinflammatory effects, an ELISA assay was performed using an LPS-induced in vitro co-culture system (Fig. 5F–H). Compared to the Sham group, the LPS-induced ICH microenvironment significantly stimulated BV2 microglia to secrete multiple pro-inflammatory cytokines, including IL-6 and TNF-α. In contrast, EW treatment reduced the expression of the pro-inflammatory cytokines IL-6 and TNF-α and significantly increased the expression of the anti-inflammatory cytokine IL-10. However, EW intervention effectively reversed the hypersecretion of these inflammatory factors, suggesting that EW exerts anti-inflammatory effects by regulating microglial activation.

In summary, both in vivo and in vitro experiments demonstrate that EW effectively inhibits excessive microglial activation, thereby mitigating neuroinflammatory responses.

### Protecting the integrity of the BBB

**Fig. 6:**
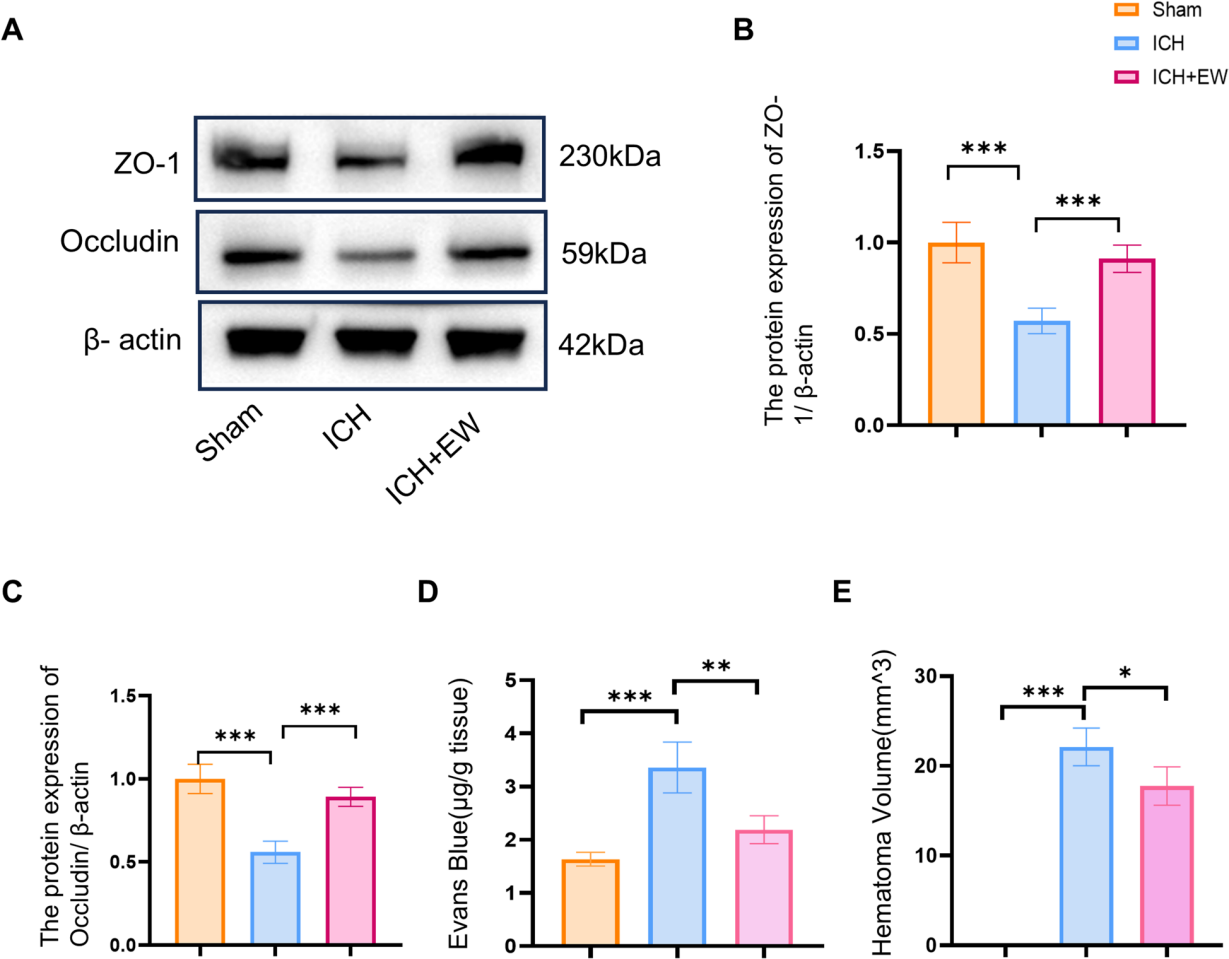
**A** Western blot evaluation of tight junction proteins ZO-1 and Occludin in brain tissues. **B**–**C** Quantitative analysis of ZO-1 and Occludin protein expression by Western blot. **D** Quantitative statistical results of Evans blue staining. **E** Quantitative analysis of hematoma volume. *p < 0.05, **p < 0.01, ***p < 0.001

Multiple pathological events following ICH, including local edema, neuroinflammation, neuronal necrosis/apoptosis, oxidative stress, and the release of clot-derived components such as hemoglobin, collectively contribute to neuronal, glial cell, and BBB damage within peri-hematomal tissue [45–48]. To assess the protective effect of EW on BBB integrity after ICH, the expression of tight junction (TJ) proteins (ZO-1 and Occludin) was examined. Western blot analysis revealed a significant downregulation of these proteins in the ICH group compared to the sham controls (*p* < 0.01, Fig. 6A–C).

To further evaluate the impact of EW on hematoma absorption and BBB integrity, hematoma volume and Evans blue extravasation in brain tissue were examined on day 3 post-ICH (Fig. 6D, E). Compared to the sham group, the ICH group exhibited substantial hematoma formation, accompanied by a significant increase in BBB permeability as indicated by Evans blue staining. Notably, EW treatment significantly reduced hematoma volume on day 3 after ICH (*p* < 0.05) and markedly decreased Evans blue extravasation (*p* < 0.01), suggesting that EW effectively preserves BBB integrity.

These results demonstrate that EW treatment promotes hematoma absorption and mitigates BBB disruption following ICH. Importantly, the reduction in hematoma volume across groups was consistent with improvements in neurological deficit scores, Evans blue extravasation, and histopathological outcomes. This suggests that the neuroprotective effects of EW may be partially attributed to its ability to promote hematoma clearance and protect the BBB, thereby alleviating secondary injury cascades such as neuroinflammation, oxidative stress, and neuronal apoptosis. Furthermore, EW directly restored the expression of TJ proteins (ZO-1 and Occludin) and reduced neuronal apoptosis in vitro, indicating that EW also exerts direct cytoprotective effects independent of hematoma clearance. Therefore, EW may exert neuroprotective effects through a dual mechanism: promoting hematoma clearance and directly mitigating secondary injury processes.

### Reduce synaptic damage

**Fig. 7:**
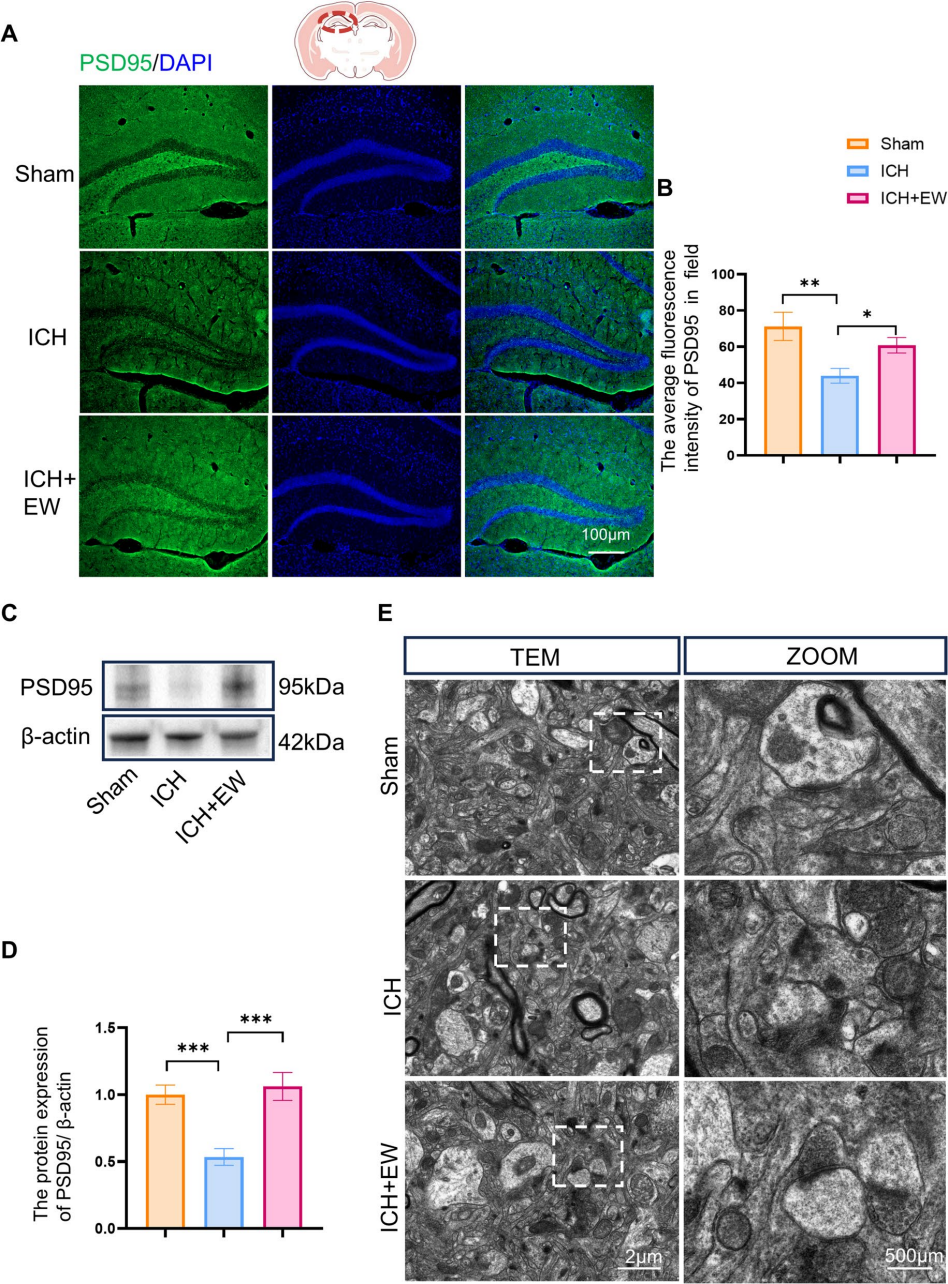
EW restores synaptic integrity following ICH. **A** Representative immunofluorescence images of hippocampal sections co-stained with PSD95 (green, postsynaptic marker) and DAPI (blue, nuclei). Scale bar: 100 μm. **B** Quantification of PSD95-positive area. **C**, **D** Western blot analysis and quantification of PSD95 expression in brain tissues. **E** Representative TEM images illustrating synaptic ultrastructure in the hippocampal region; the boxed area is magnified to show detailed synaptic morphology. Scale bars: 2 μm (overview), 500 nm (enlarged). *p < 0.05, **p < 0.01, ***p < 0.001

During the pathological process of ICH, hemoglobin degradation products, including iron ions, and inflammatory factors such as TNF-α and IL-1β, released from the hematoma, can damage the expression and localization of postsynaptic density protein 95 (PSD95) through oxidative stress and calcium overload. This disrupts synaptic structures. PSD95 expression is closely correlated with synapse number, morphological integrity, and neural signaling function. In the ICH mouse model, PSD95 immunofluorescence staining revealed sparse and scattered PSD95 signals in the hippocampus of mice in the ICH group, with some areas showing no fluorescence. In contrast, the EW treatment group exhibited significantly enhanced intensity and density of PSD95-positive signals in the hippocampus (Fig. 7A, B). Western blot quantification confirmed that EW effectively reversed the ICH-induced downregulation of PSD95 expression (Fig. 7C, D).

To further verify these findings, the synaptic ultrastructure was examined using electron microscopy. The results showed that synaptic structures in the normal control group were clearly identifiable, with a uniformly thick postsynaptic dense region and numerous synaptic vesicles densely arranged in the presynaptic membrane. In the ICH group, a significant reduction in synaptic density was observed, and the remaining synapses exhibited severe structural impairments. These included widened and irregular synaptic spaces, thinner postsynaptic dense regions, fewer synaptic vesicles in the presynaptic membrane, and disordered vesicle distribution. In contrast, the synaptic structure of the EW-treated mice closely resembled that of the normal control group (Fig. 7E).

### Extracellular transcriptionomics changes in EW

**Fig. 8:**
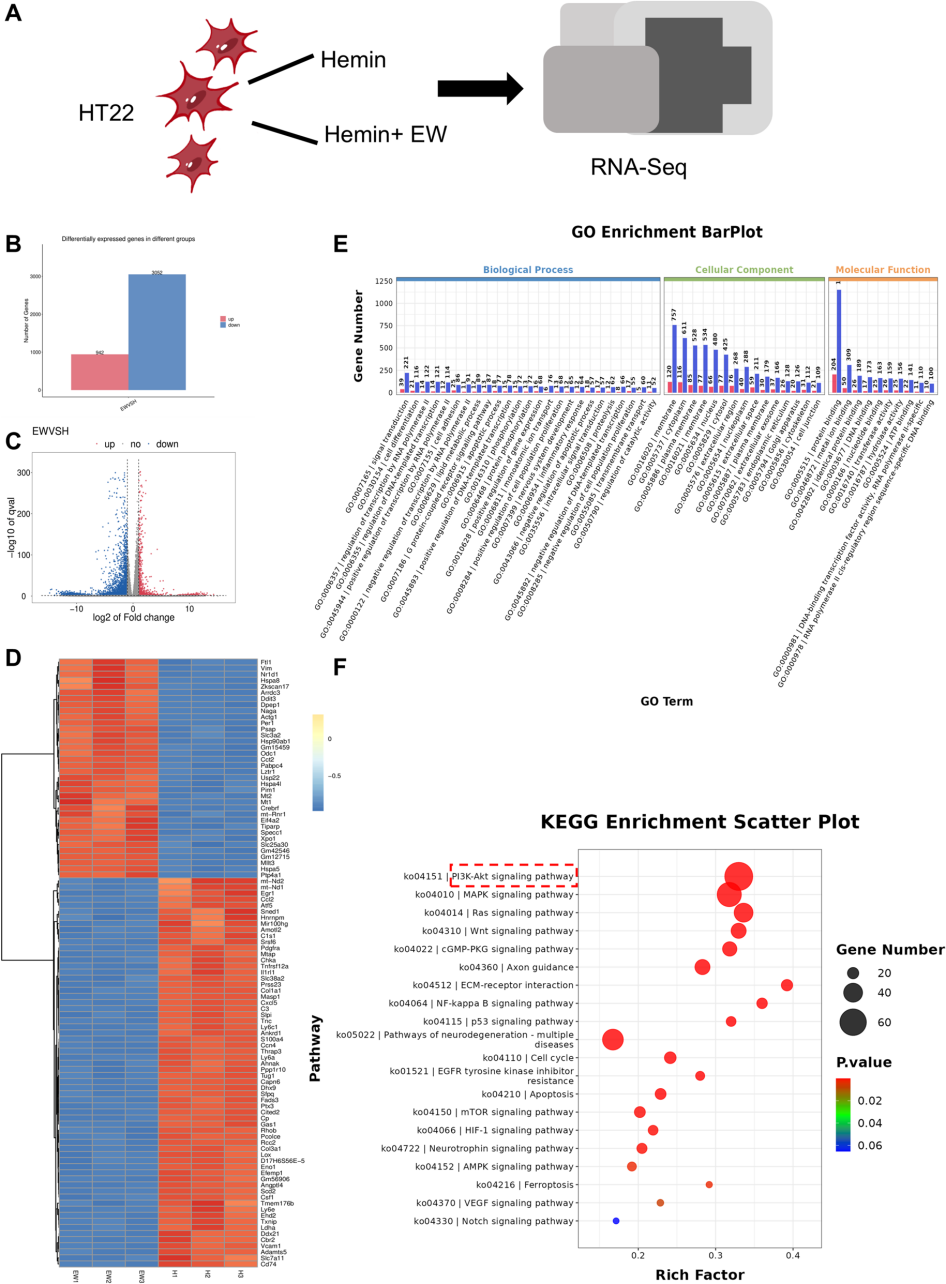
EW activates the PI3K/AKT signaling pathway following ICH. **A** Schematic overview of the transcriptome sequencing workflow. **B**–**D** Visualization of differentially expressed genes: **B** MA plot, **C** volcano plot, and **D** hierarchical clustering heatmap. **E** Gene Ontology (GO) enrichment analysis of biological processes. **F** KEGG pathway enrichment analysis highlighting the PI3K/AKT signaling pathway

To identify the key targets underlying EW's protective effects in the Hemin-induced ICH model, RNA-seq analysis was conducted on HT22 cells from both the Hemin and Hemin + EW groups (Fig. 8A). The results confirmed that EW mitigates hemin-induced cellular damage and exerts neuroprotective effects. DEGs were identified by comparing the Hemin and Hemin + EW groups in HT22 cells (Fig. 8B), revealing 942 upregulated genes and 3,052 downregulated genes. GO enrichment analysis showed that EW's neuroprotective effects involved key biological processes such as chemical synaptic transmission, synaptic signaling, intercellular signaling, and nervous system development (Fig. 8C–E). KEGG pathway enrichment analysis indicated that important signaling pathways, including those involved in cell proliferation, axon guidance, neuroactive ligand-receptor interactions, and neurotrophic factors, were modulated by EW. By regulating these pathways, EW appears to orchestrate a multifaceted neuroprotective response, preserving neuronal function while counteracting hemin-induced injury and promoting neurogenesis and functional restoration.

Further KEGG enrichment analysis revealed the PI3K/AKT signaling pathway as significantly enriched in the Hemin + EW group compared to the Hemin group (Fig. 8F). The PI3K/AKT pathway transduces extracellular signals to regulate cell survival, growth, and angiogenesis, involving PI3K-mediated phosphorylation of its downstream effector, the serine/threonine kinase AKT [49]. Previous studies have highlighted the PI3K/AKT signaling pathway's role in neuroprotection, including its ability to counteract neuroinflammation, oxidative stress, and neuronal apoptosis [50, 51]. Based on these findings, it is hypothesized that EW modulates neuronal proliferation and apoptosis after ICH via the PI3K/AKT pathway, and further experiments were conducted to validate this mechanism.

### Mechanism pathway verification

In the cellular regulatory network governing survival and apoptosis, the PI3K/AKT pathway plays a pivotal role. The Bcl-2 family of proteins acts as key effectors downstream of this pathway, forming the core axis of the "signal pathway-apoptosis regulatory protein" system. This axis is critical for maintaining cellular homeostasis and regulating programmed cell death in neurons following cerebral hemorrhage. Building on previous findings, RNA-seq analysis revealed that EW treatment significantly enhances the PI3K/AKT signaling pathway, which subsequently modulates Bax activity through phosphorylation.

**Fig. 9:**
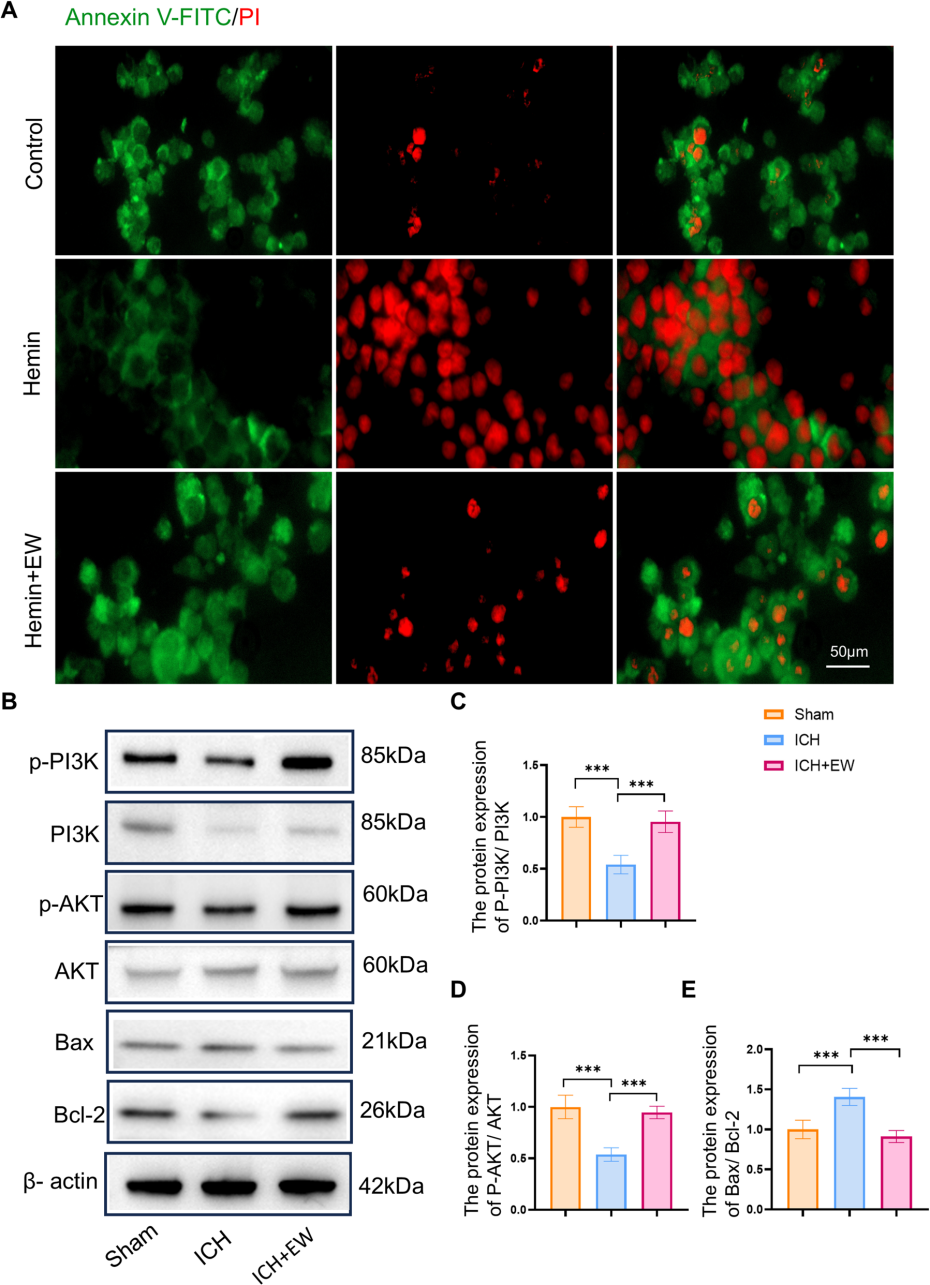
EW attenuates neuronal apoptosis via PI3K/AKT signaling activation. **A** Representative images of Annexin V-FITC/PI staining in primary neurons: green (Annexin V-FITC, early apoptosis), red (PI, necrotic/late apoptotic cells). Scale bar: 50 μm. **B**–**E** Western blot analysis of PI3K/AKT pathway activation (p-PI3K, PI3K, p-AKT, AKT) and apoptosis-related proteins (Bax, Bcl-2) in brain tissues. *p < 0.05, **p < 0.01, ***p < 0.001

Primary neurons were treated with hemin in vitro, followed by Annexin V-FITC/PI staining to assess EW's impact on apoptotic processes. Annexin V-FITC-positive cells exhibited green fluorescence on their cell membranes, while PI-positive cells appeared red. Early apoptotic cells did not stain with PI, while necrotic or late apoptotic cells displayed red cytoplasm with a green membrane. After co-incubating EW with hemin for 24 h, reduced red fluorescence was observed, indicating that EW mitigates neuronal apoptosis (Fig. 9A). To further confirm the effect of EW on Bax activity, Western blot analysis was conducted on brain tissue surrounding the hematoma in mice. The in vivo results corroborated the in vitro data, showing that EW modulated apoptosis-related proteins by reducing Bax levels and increasing Bcl-2 expression (Fig. 9B–E).

**Fig. 10:**
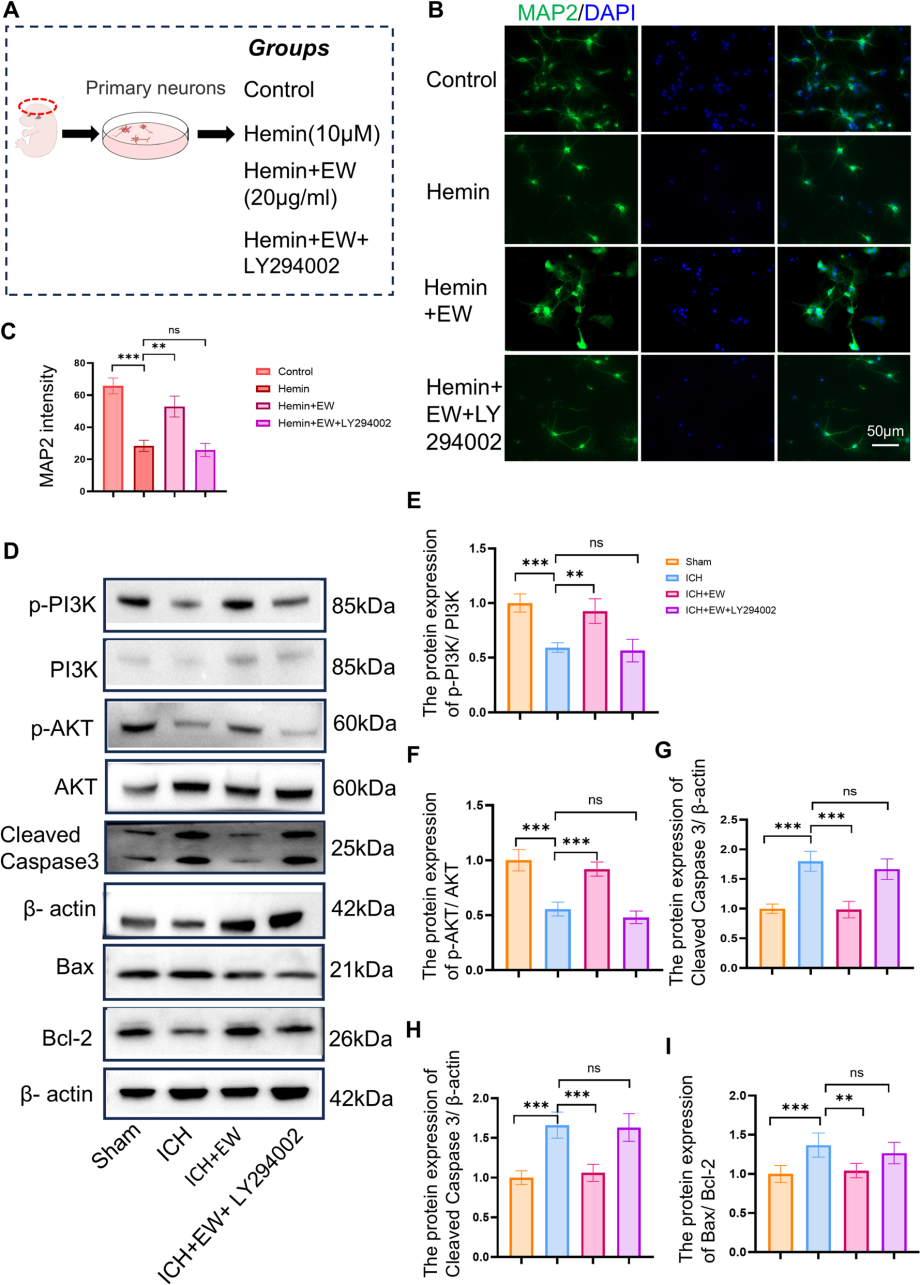
EW activates the PI3K/AKT signaling pathway to suppress neuronal apoptosis. **A** Schematic of the primary neuron-based validation experiment. **B** Representative immunofluorescence images of primary neurons stained for MAP2 (green, neuronal marker) and DAPI (blue, nuclei) under hemin challenge with or without EW treatment. **C** Quantification of MAP2-positive area. **D** Proposed mechanism: EW promotes PI3K/AKT pathway activation, which modulates downstream effectors Bax and Bcl-2 to attenuate apoptosis. **E**–**I** Western blot quantification of p-PI3K, PI3K, p-AKT, AKT, Cleaved Caspase-3, Bax, and Bcl-2 protein levels in primary neurons. *p < 0.05, **p < 0.01, ***p < 0.001

To explore the underlying mechanism of EW's neuroprotective effects against ICH, and based on the previous in vivo data showing that EW upregulates the anti-apoptotic protein Bcl-2, this study further investigated whether EW modulates neuronal apoptosis through the PI3K/AKT signaling pathway. Our results revealed that EW treatment promoted neuronal survival and growth, whereas the PI3K inhibitor reversed these beneficial effects and suppressed neuronal protein expression. Subsequent in vivo assays indicated that the PI3K/AKT pathway serves as a key upstream regulator mediating EW-induced modulation of Bcl-2 expression. Inhibition of PI3K directly affected the protein levels of both Bax and Bcl-2 (Fig. 10A–C). Furthermore, the Bax/Bcl-2 ratio was significantly elevated in the ICH group, but decreased substantially following EW treatment, shifting the balance toward an anti-apoptotic phenotype. Concurrently, Cleaved Caspase-3 activity was reduced, and the number of apoptotic neurons decreased, ultimately alleviating ICH-induced neurological deficits (Fig. 10D).

**Fig. 11:**
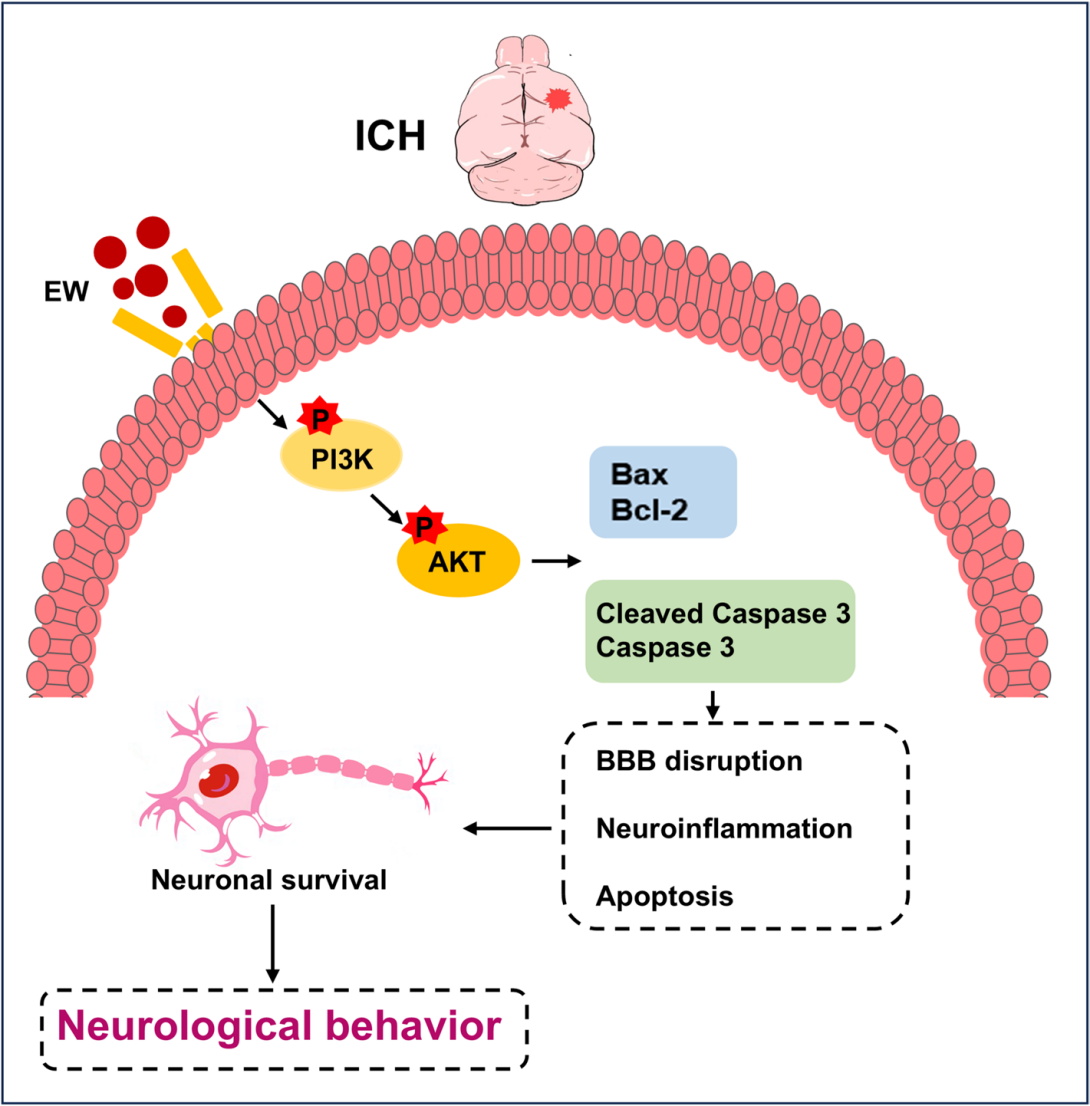
Proposed mechanism of EW-mediated neuroprotection after ICH. This schematic illustrates the multifaceted protective effects of EW in the ICH brain: suppression of neuroinflammation through microglial polarization, reduction of oxidative stress and neuronal apoptosis via PI3K/AKT pathway activation, preservation of blood–brain barrier integrity, and restoration of synaptic structure and function

These results demonstrate that EW activates Bcl-2 via the PI3K/AKT pathway, enhancing neuronal resistance to apoptosis and ameliorating functional impairment in ICH mice. The underlying mechanisms are summarized in Fig. 11.

## Discussion

Neuronal injury and neuroinflammation are key pathophysiological contributors to the poor prognosis following ICH [52]. The present study demonstrated the neuroprotective effects of EW in ICH mice through comprehensive behavioral assessments. Primary injury after ICH is caused by direct hematoma compression, while secondary injury involves a cascade of pathophysiological events activated by components released from the hematoma [53]. During the progression of secondary injury, hematoma-derived factors trigger a pathological cascade that includes axonal demyelination, neuronal degeneration, abnormal synaptogenesis, and macrophage infiltration [54]. As such, secondary brain injury (SBI) in ICH can lead to irreversible damage and exacerbate neurological deficits [55]. EW significantly mitigated neuronal damage, with immunofluorescence staining revealing an uneven MAP2 fluorescence distribution in the ICH group, indicating pronounced lesion morphology. In contrast, the EW group showed a recovery in fluorescence distribution, becoming more uniform. Western blot analysis further confirmed a significant increase in MAP2 expression after EW treatment, validating the reduction of neuronal injury associated with ICH. In an in vitro model of neuron injury induced by hemin, EW treatment significantly reduced primary neuron cell death. The anti-apoptotic effect of EW on primary neurons was confirmed through Annexin V-FITC/PI staining, which was consistent in both in vitro and in vivo experiments, demonstrating EW's neuroprotective properties.

The BBB, composed of cerebral microvascular endothelial cells, TJ complexes, astrocytes, and pericytes, serves as a protective barrier that maintains the integrity of the CNS by blocking external pathogens and regulating the exchange of substances with the peripheral blood system [56]. Promoting hematoma resolution is a critical therapeutic target for improving ICH outcomes, as BBB breakdown initiates a cascade of events that hinder neurological recovery [57]. EW treatment reduced GFAP expression and alleviated the morphological activation of astrocytes, suggesting an inhibitory effect on astrocyte activation. The hippocampus, a key brain region linked to learning and memory, is particularly sensitive to astrocyte overactivation, which negatively affects neuronal function. The functional triad of astrocytes, endothelial cells, and pericytes is crucial in maintaining BBB integrity through dynamically regulated signaling mechanisms [58]. In the present study, ICH-induced degradation of key TJ proteins (Occludin and ZO-1) was observed, leading to capillary damage and BBB failure. Maintaining TJ integrity is essential for minimizing BBB damage, promoting neurological recovery, and reducing SBI after ICH [59].

Neuroinflammation is central to the pathological evolution of ICH, triggering oxidative stress, excitotoxicity, and cell death through coordinated signaling pathway activation [60]. EW's inhibitory effect on neuroinflammation plays a pivotal role in improving neuronal function. Numerous studies have shown that the inflammatory cascade post-ICH exacerbates the hematoma mass effect, accelerates perihematoma edema, induces neuronal apoptosis, and impairs neurological outcomes [7, 61]. Following ICH, a pathological triad of microglial/macrophage activation, neutrophil recruitment, and proinflammatory cytokine release amplifies neuroinflammatory responses and neuronal apoptosis [7]. In the present study, EW significantly reduced the fluorescence intensity of iNOS and the expression of the proinflammatory cytokine IL-6 following LPS stimulation. EW effectively alleviates the neuroinflammatory response by modulating microglial phenotypic shifts toward an anti-inflammatory state and suppressing inflammatory signaling pathways, thereby mitigating further neuronal injury driven by neuroinflammation [43, 44].

The neuroinflammatory response following ICH involves intricate interactions among microglia, astrocytes, and neurons. Our findings demonstrate that EW effectively inhibits microglial activation (Fig. 4E, F) and reduces the secretion of pro-inflammatory cytokines, including IL-6, and TNF-α (Fig. 5F–H). Since these cytokines are known to promote astrocyte reactivity and neuronal apoptosis [62–64], the observed reduction in astrocyte activation (Fig. 4A–D) and the improvement in neurological outcomes in the EW-treated group may be partially attributed to the suppression of microglia-mediated inflammatory signaling. These results suggest that EW may exert its neuroprotective effects by modulating the interactions between microglia, astrocytes, and neurons; however, further investigation is needed to fully elucidate the precise molecular mechanisms underlying this regulation.

RNA-seq analysis provided insights into the molecular mechanisms by which EW exerts its neuroprotective effects. DEGs were found to be strongly associated with key neuronal functions, notably chemical synaptic transmission and synaptic signal transduction. KEGG enrichment analysis revealed that EW modulates various signaling pathways, including axon guidance, neuroactive ligand–receptor interactions, and neurotrophic factors. Among the most significantly enriched pathways, the PI3K/AKT pathway emerged as a central mediator of EW's protective effects, including attenuated brain damage, suppressed neuroinflammation, and reduced neuronal apoptosis [65]. Our results highlight the PI3K/AKT-mediated upregulation of Bcl-2 as a key mechanism through which EW reduces apoptosis and improves neurological outcomes, providing molecular-level evidence for its therapeutic efficacy.

Notably, the KEGG enrichment scatter plot revealed the PI3K/AKT pathway as the most significantly enriched core pathway, while the MAPK and NF-κB pathways were also prominently enriched, suggesting potential crosstalk between these pathways. Although our findings support the involvement of the PI3K/AKT pathway in the neuroprotective effects of EW, definitive mechanistic conclusions cannot yet be drawn. In this study, a non-selective PI3K inhibitor (LY294002) was used, which may carry a risk of off-target effects. Additionally, the upstream regulators and specific isoforms involved in this pathway remain to be identified. Therefore, the current results should be viewed as supportive evidence rather than conclusive mechanistic proof. Future studies employing more specific intervention strategies and genetic approaches are needed to validate and refine the underlying molecular mechanisms, providing a stronger theoretical foundation for the clinical application of EW in the treatment of ICH.

In this study, the HT22 mouse hippocampal neuronal cell line was utilized as an in vitro model. HT22 cells are a widely used and valuable tool in neurobiology, particularly suited for investigating neurotoxicity induced by neuronal injury, oxidative stress, and various forms of programmed cell death associated with acute neurological injury [66, 67]. The use of this cell line offers practical advantages, including cellular homogeneity, genetic stability, and ease of culture, which facilitate a detailed exploration of specific pathways at the molecular level.

Nevertheless, this study has several limitations that should be considered when interpreting the findings and assessing their generalizability. The experiments were conducted exclusively in young adult male C57BL/6 J mice, excluding female mice, older animals, and models with comorbidities. While using a cohort of male mice at a single age minimizes potential confounding factors such as fluctuations in sex hormone levels, age-related physiological decline, and underlying comorbidities—thereby enhancing the stability and reproducibility of the preliminary mechanistic results—this design significantly limits the external validity and broader applicability of our conclusions. As a result, the findings may not fully capture the complexity of pathophysiological processes across different sexes, age groups, or individuals with pre-existing conditions.

## Conclusion

The limitations of this study highlight the need for further research to deepen our understanding of the underlying mechanisms. Specifically, the precise molecular mechanisms through which EW activates the PI3K/AKT signaling pathway remain to be elucidated. From a clinical perspective, investigating the multi-target mechanisms of EW's action will provide stronger scientific evidence for its potential clinical use, positioning EW as a promising adjunctive treatment for ICH. For patients with ICH, especially during the recovery phase, EW may facilitate the recovery of motor and cognitive functions by improving neurological outcomes, reducing inflammation, and protecting neurons, ultimately enhancing quality of life. Moreover, since EW is administered orally, it offers greater convenience compared to injectable drugs, potentially improving patient compliance and promoting its widespread clinical adoption.

Overall, EW demonstrates neuroprotective effects through mechanisms such as neuronal protection, reduction of neuronal apoptosis, inhibition of astrocyte activation, suppression of neuroinflammation, and regulation of microglial polarization.

## Supplementary Information

Additional file 1.

## Data Availability

The data that support the findings of this study are available from the corresponding author upon reasonable request.

## Abbreviations

ICH: Intracerebral hemorrhage
EW: Eerdun Wurile
RNA-seq: RNA sequencing
EDA: Edaravone injection
MWM: Morris water maze
EPM: Elevated plus maze
Iba-1: Ionized calcium-binding adapter molecule 1
LPS: Lipopolysaccharide
GFAP: Glial fibrillary acidic protein
BBB: Blood–brain barrier
CNS: Central nervous system
PSD95: Postsynaptic density protein 95
PI3K: Phosphatidylinositol 3-kinase
AKT: Protein kinase B

## References

[1] Puy L, Parry-Jones AR, Sandset EC, Dowlatshahi D, Ziai W, Cordonnier C. Intracerebral haemorrhage. Nat Rev Dis Primers. 2023;9(1):14. 10.1038/s41572-023-00424-7. 36928219 10.1038/s41572-023-00424-7

[2] Wang Y, Tian M, Tan J, Pei X, Lu C, Xin Y, et al. Irisin ameliorates neuroinflammation and neuronal apoptosis through integrin αVβ5/AMPK signaling pathway after intracerebral hemorrhage in mice. J Neuroinflammation. 2022;19:82. 10.1186/s12974-022-02438-6. 35392928 10.1186/s12974-022-02438-6 PMC8988353

[3] Al-Kawaz MN, Hanley DF, Ziai W. Advances in therapeutic approaches for spontaneous intracerebral hemorrhage. Neurotherapeutics. 2020;17:1757–67. 10.1007/s13311-020-00902-w. 32720246 10.1007/s13311-020-00902-w PMC7851203

[4] Lattanzi S, Di Napoli M, Ricci S, Divani AA. Matrix metalloproteinases in acute intracerebral hemorrhage. Neurotherapeutics. 2020;17:484–96. 10.1007/s13311-020-00839-0. 31975152 10.1007/s13311-020-00839-0 PMC7283398

[5] Duan T, Li L, Yu Y, Li T, Han R, Sun X, et al. Traditional Chinese medicine use in the pathophysiological processes of intracerebral hemorrhage and comparison with conventional therapy. Pharmacol Res. 2022;179:106200. 10.1016/j.phrs.2022.106200. 35367344 10.1016/j.phrs.2022.106200

[6] Zhou Y, Wang Y, Wang J, Anne Stetler R, Yang QW. Inflammation in intracerebral hemorrhage: from mechanisms to clinical translation. Prog Neurobiol. 2014;115:25–44. 10.1016/j.pneurobio.2013.11.003. 24291544 10.1016/j.pneurobio.2013.11.003

[7] Chen S, Peng J, Sherchan P, Ma Y, Xiang S, Yan F, et al. TREM2 activation attenuates neuroinflammation and neuronal apoptosis via PI3K/Akt pathway after intracerebral hemorrhage in mice. J Neuroinflammation. 2020;17:168. 10.1186/s12974-020-01853-x. 32466767 10.1186/s12974-020-01853-x PMC7257134

[8] Liu J, Liu L, Wang X, Jiang R, Bai Q, Wang G. Microglia: a double-edged sword in intracerebral hemorrhage from basic mechanisms to clinical research. Front Immunol. 2021;12:675660. 10.3389/fimmu.2021.675660. 34025674 10.3389/fimmu.2021.675660 PMC8135095

[9] Ziai WC. Hematology and inflammatory signaling of intracerebral hemorrhage. Stroke. 2013;44:S74-78. 10.1161/strokeaha.111.000662. 23709738 10.1161/STROKEAHA.111.000662 PMC12054399

[10] Wu X, Luo J, Liu H, Cui W, Guo K, Zhao L, et al. Recombinant adiponectin peptide ameliorates brain injury following intracerebral hemorrhage by suppressing astrocyte-derived inflammation via the inhibition of Drp1-mediated mitochondrial fission. Transl Stroke Res. 2020;11:924–39. 10.1007/s12975-019-00768-x. 31902083 10.1007/s12975-019-00768-x

[11] Bian L, Zhang J, Wang M, Keep RF, Xi G, Hua Y. Intracerebral hemorrhage-induced brain injury in rats: the role of extracellular peroxiredoxin 2. Transl Stroke Res. 2020;11:288–95. 10.1007/s12975-019-00714-x. 31273681 10.1007/s12975-019-00714-x PMC6942235

[12] Li W, Chopp M, Zacharek A, Yang W, Chen Z, Landschoot-Ward J, et al. SUMO1 deficiency exacerbates neurological and cardiac dysfunction after intracerebral hemorrhage in aged mice. Transl Stroke Res. 2021;12:631–42. 10.1007/s12975-020-00837-6. 32761461 10.1007/s12975-020-00837-6

[13] Shao A, Zhu Z, Li L, Zhang S, Zhang J. Emerging therapeutic targets associated with the immune system in patients with intracerebral haemorrhage (ICH): from mechanisms to translation. EBioMedicine. 2019;45:615–23. 10.1016/j.ebiom.2019.06.012. (**13.**). 31208948 10.1016/j.ebiom.2019.06.012 PMC6642355

[14] Zheng Y, Li R, Fan X. Targeting oxidative stress in intracerebral hemorrhage: prospects of the natural products approach. Antioxidants (Basel). 2022. 10.3390/antiox11091811. 36139885 10.3390/antiox11091811 PMC9495708

[15] Gaowa S, Bao N, Da M, Qiburi Q, Ganbold T, Chen L, et al. Traditional Mongolian medicine Eerdun Wurile improves stroke recovery through regulation of gene expression in rat brain. J Ethnopharmacol. 2018;222:249–60. 10.1016/j.jep.2018.05.011. 29758340 10.1016/j.jep.2018.05.011

[16] Qiao Y, Li H, Li Y, Su E, Wang Z, Che L, et al. Study on the mechanism of Eerdun Wurile’s effects on post-operative cognitive dysfunction by the TLR4/NF-κB pathway. Mol Neurobiol. 2023;60:7274–84. 10.1007/s12035-023-03537-y. 37548853 10.1007/s12035-023-03537-y PMC10657789

[17] Liu T, Wang W, Li X, Chen Y, Mu F, Wen A, et al. Advances of phytotherapy in ischemic stroke targeting PI3K/Akt signaling. Phytother Res. 2023;37:5509–28. 10.1002/ptr.7994. 37641491 10.1002/ptr.7994

[18] Zhang G, Lu J, Zheng J, Mei S, Li H, Zhang X, et al. Spi1 regulates the microglial/macrophage inflammatory response via the PI3K/AKT/mTOR signaling pathway after intracerebral hemorrhage. Neural Regen Res. 2024;19:161–70. 10.4103/1673-5374.375343. 37488863 10.4103/1673-5374.375343 PMC10479839

[19] Deng S, Jin P, Sherchan P, Liu S, Cui Y, Huang L, et al. Recombinant CCL17-dependent CCR4 activation alleviates neuroinflammation and neuronal apoptosis through the PI3K/AKT/Foxo1 signaling pathway after ICH in mice. J Neuroinflammation. 2021;18:62. 10.1186/s12974-021-02112-3. 33648537 10.1186/s12974-021-02112-3 PMC7923481

[20] Zhao M, Gao J, Zhang Y, Jiang X, Tian Y, Zheng X, et al. Elevated miR-29a contributes to axonal outgrowth and neurological recovery after intracerebral hemorrhage via targeting PTEN/PI3K/Akt pathway. Cell Mol Neurobiol. 2021;41:1759–72. 10.1007/s10571-020-00945-9. 32889668 10.1007/s10571-020-00945-9 PMC11444011

[21] Yan J, Zhang Y, Wang L, Li Z, Tang S, Wang Y, et al. TREM2 activation alleviates neural damage via Akt/CREB/BDNF signalling after traumatic brain injury in mice. J Neuroinflammation. 2022;19:289. 10.1186/s12974-022-02651-3. 36463233 10.1186/s12974-022-02651-3 PMC9719652

[22] Naranmandula S. Effect of Mongolian medicine Eerdun Wurile on the Nrf2/HO-1 signaling pathway in brain tissue of rats with ischemic vertigo. World J Integr Trad West Med. 2025;20:1074–9. 10.13935/j.cnki.sjzx.250602.

[23] Zhao C, Hu Y. Effect of Mongolian medicine Eerdun Wurile on cognitive dysfunction in vascular dementia rats. Chin J Ethnomed Ethnopharm. 2024;30:43–5. 10.16041/j.cnki.cn15-1175.2024.09.015.

[24] Wu X, Zhang Y, Zhang Y, Xia L, Yang Y, Wang P, et al. MST4 attenuates NLRP3 inflammasome-mediated neuroinflammation and affects the prognosis after intracerebral hemorrhage in mice. Brain Res Bull. 2021;177:31–8. 10.1016/j.brainresbull.2021.09.006. 34534636 10.1016/j.brainresbull.2021.09.006

[25] Lv Z, Che L, Du Y, Yu J, Su E, Liu H, et al. Mechanism of Mongolian medicine Eerdun Wurile in improving postoperative cognitive dysfunction through activation of the PI3K signaling pathway. Front Neurosci. 2021;15:769759. 10.3389/fnins.2021.769759. 35095392 10.3389/fnins.2021.769759 PMC8798410

[26] Wang C, Cheng F, Han Z, Yan B, Liao P, Yin Z, et al. Human-induced pluripotent stem cell–derived neural stem cell exosomes improve blood–brain barrier function after intracerebral hemorrhage by activating astrocytes via PI3K/AKT/MCP-1 axis. Neural Regen Res. 2025;20:518–32. 10.4103/nrr.Nrr-d-23-01889. 38819064 10.4103/NRR.NRR-D-23-01889 PMC11317932

[27] Tong LS, Shao AW, Ou YB, Guo ZN, Manaenko A, Dixon BJ, et al. Recombinant Gas6 augments Axl and facilitates immune restoration in an intracerebral hemorrhage mouse model. J Cereb Blood Flow Metab. 2017;37:1971–81. 10.1177/0271678x16658490. 27389179 10.1177/0271678X16658490 PMC5464693

[28] Chen J, Li Y, Wang L, Zhang Z, Lu D, Lu M, et al. Therapeutic benefit of intravenous administration of bone marrow stromal cells after cerebral ischemia in rats. Stroke. 2001;32:1005–11. 10.1161/01.str.32.4.1005. 11283404 10.1161/01.str.32.4.1005

[29] Chen J, Sanberg PR, Li Y, Wang L, Lu M, Willing AE, et al. Intravenous administration of human umbilical cord blood reduces behavioral deficits after stroke in rats. Stroke. 2001;32:2682–8. 10.1161/hs1101.098367. 11692034 10.1161/hs1101.098367

[30] Ding R, Feng L, He L, Chen Y, Wen P, Fu Z, et al. Peroxynitrite decomposition catalyst prevents matrix metalloproteinase-9 activation and neurovascular injury after hemoglobin injection into the caudate nucleus of rats. Neuroscience. 2015;297:182–93. 10.1016/j.neuroscience.2015.03.065. 25849612 10.1016/j.neuroscience.2015.03.065

[31] Li Y, Yang W, Tang Y, Du S, Ye Y, Xu B, et al. Ectomesenchymal stem cell transplantation induces microglial polarization and anti-inflammatory IL-10 secretion via NF-κB and MAPK pathways to mitigate brain injury post-cerebral hemorrhage. Neurochem Res. 2025;51:20. 10.1007/s11064-025-04633-2. 41420699 10.1007/s11064-025-04633-2

[32] Hong L, Zhuo T, Jing S. Silencing of METTL3 inhibits m6A methylation of NEK7 to suppress pyrolysis in an HT-22 cell-based model of intracerebral hemorrhage. Brain Res. 2024;1831:148828. 10.1016/j.brainres.2024.148828. 38408556 10.1016/j.brainres.2024.148828

[33] Zhang X, Wu Q, Zhang Q, Lu Y, Liu J, Li W, et al. Resveratrol attenuates early brain injury after experimental subarachnoid hemorrhage via inhibition of NLRP3 inflammasome activation. Front Neurosci. 2017;11:611. 10.3389/fnins.2017.00611. (**29.**). 29163015 10.3389/fnins.2017.00611 PMC5675880

[34] Jing J, Chen S, Wu X, Yang J, Liu X, Wang J, et al. Recombinant tissue plasminogen activator protects neurons after intracerebral hemorrhage through activating the PI3K/AKT/mTOR pathway. Neural Regen Res. 2026;21:1574–85. 10.4103/nrr.Nrr-d-23-01953. 39104167 10.4103/NRR.NRR-D-23-01953 PMC12407559

[35] Lei P, Li Z, Hua Q, Song P, Gao L, Zhou L, et al. Ursolic acid alleviates neuroinflammation after intracerebral hemorrhage by mediating microglial pyroptosis via the NF-κB/NLRP3/GSDMD pathway. Int J Mol Sci. 2023. 10.3390/ijms241914771. 37834220 10.3390/ijms241914771 PMC10572659

[36] Zhang S, Hu ZW, Luo HY, Mao CY, Tang MB, Li YS, et al. AAV/BBB-mediated gene transfer of CHIP attenuates brain injury following experimental intracerebral hemorrhage. Transl Stroke Res. 2020;11:296–309. 10.1007/s12975-019-00715-w. 31325153 10.1007/s12975-019-00715-w

[37] Sahraeian SME, Mohiyuddin M, Sebra R, Tilgner H, Afshar PT, Au KF, et al. Gaining comprehensive biological insight into the transcriptome by performing a broad-spectrum RNA-seq analysis. Nat Commun. 2017;8:59. 10.1038/s41467-017-00050-4. 28680106 10.1038/s41467-017-00050-4 PMC5498581

[38] Love MI, Huber W, Anders S. Moderated estimation of fold change and dispersion for RNA-seq data with DESeq2. Genome Biol. 2014;15:550. 10.1186/s13059-014-0550-8. 25516281 10.1186/s13059-014-0550-8 PMC4302049

[39] Lim TC, Mandeville E, Weng D, Wang LS, Kurisawa M, Leite-Morris K, et al. Hydrogel-based therapy for brain repair after intracerebral hemorrhage. Transl Stroke Res. 2020;11:412–7. 10.1007/s12975-019-00721-y. 31432328 10.1007/s12975-019-00721-y

[40] Peressotti S, Koehl GE, Goding JA, Green RA. Self-assembling hydrogel structures for neural tissue repair. ACS Biomater Sci Eng. 2021;7:4136–63. 10.1021/acsbiomaterials.1c00030. 33780230 10.1021/acsbiomaterials.1c00030 PMC8441975

[41] Puy L, Parry-Jones AR, Sandset EC, Dowlatshahi D, Ziai W, Cordonnier C. Intracerebral haemorrhage. Nat Rev Dis Primers. 2023;9:14. 10.1038/s41572-023-00424-7. 36928219 10.1038/s41572-023-00424-7

[42] Magid-Bernstein J, Girard R, Polster S, Srinath A, Romanos S, Awad IA, et al. Cerebral hemorrhage: pathophysiology, treatment, and future directions. Circ Res. 2022;130:1204–29. 10.1161/circresaha.121.319949. 35420918 10.1161/CIRCRESAHA.121.319949 PMC10032582

[43] Yu W, Che C, Yang Y, Zhao Y, Liu J, Chen A, et al. Bioactive self-assembled nanoregulator enhances hematoma resolution and inhibits neuroinflammation in the treatment of intracerebral hemorrhage. Adv Sci. 2025;12:e2408647. 10.1002/advs.202408647. 10.1002/advs.202408647 PMC11714160 39520083

[44] Wang T, Lei H, Li X, Yang N, Ma C, Li G, et al. Magnetic targeting nanocarriers combined with focusing ultrasound for enhanced intracerebral hemorrhage therapy. Small. 2023;19:e2206982. 10.1002/smll.202206982. 36703527 10.1002/smll.202206982

[45] Ma Q, Huang B, Khatibi N, Rolland W 2nd, Suzuki H, Zhang JH, et al. PDGFR-α inhibition preserves blood-brain barrier after intracerebral hemorrhage. Ann Neurol. 2011;70:920–31. 10.1002/ana.22549. 22190365 10.1002/ana.22549 PMC3405848

[46] Selim M, Sheth KN. Perihematoma edema: a potential translational target in intracerebral hemorrhage? Transl Stroke Res. 2015;6:104–6. 10.1007/s12975-015-0389-7. 25693976 10.1007/s12975-015-0389-7 PMC4359064

[47] Keep RF, Hua Y, Xi G. Intracerebral haemorrhage: mechanisms of injury and therapeutic targets. Lancet Neurol. 2012;11:720–31. 10.1016/s1474-4422(12)70104-7. 22698888 10.1016/S1474-4422(12)70104-7 PMC3884550

[48] Stockwell BR, Friedmann Angeli JP, Bayir H, Bush AI, Conrad M, Dixon SJ, et al. Ferroptosis: a regulated cell death nexus linking metabolism, redox biology, and disease. Cell. 2017;171:273–85. 10.1016/j.cell.2017.09.021. 28985560 10.1016/j.cell.2017.09.021 PMC5685180

[49] Zhu M, Li D, Wu Y, Huang X, Wu M. TREM-2 promotes macrophage-mediated eradication of *Pseudomonas**aeruginosa* via a PI3K/Akt pathway. Scand J Immunol. 2014;79:187–96. 10.1111/sji.12148. 24383713 10.1111/sji.12148

[50] He H, Liu W, Zhou Y, Liu Y, Weng P, Li Y, et al. Sevoflurane post-conditioning attenuates traumatic brain injury-induced neuronal apoptosis by promoting autophagy via the PI3K/AKT signaling pathway. Drug Des Devel Ther. 2018;12:629–38. 10.2147/dddt.S158313. 29606856 10.2147/DDDT.S158313 PMC5868589

[51] Tu XK, Zhang HB, Shi SS, Liang RS, Wang CH, Chen CM, et al. 5-LOX inhibitor zileuton reduces inflammatory reaction and ischemic brain damage through the activation of PI3K/Akt signaling pathway. Neurochem Res. 2016;41:2779–87. 10.1007/s11064-016-1994-x. 27380038 10.1007/s11064-016-1994-x

[52] Rost NS, Brodtmann A, Pase MP, van Veluw SJ, Biffi A, Duering M, et al. Post-stroke cognitive impairment and dementia. Circ Res. 2022;130:1252–71. 10.1161/circresaha.122.319951. 35420911 10.1161/CIRCRESAHA.122.319951

[53] Zille M, Farr TD, Keep RF, Römer C, Xi G, Boltze J. Novel targets, treatments, and advanced models for intracerebral haemorrhage. EBioMedicine. 2022;76:103880. 10.1016/j.ebiom.2022.103880. 35158309 10.1016/j.ebiom.2022.103880 PMC8850756

[54] Seiffge DJ, Anderson CS. 49.Treatment for intracerebral hemorrhage: dawn of a new era. Int J Stroke. 2024;19:482–9. 10.1177/17474930241250259. 38803115 10.1177/17474930241250259

[55] Cordonnier C, Demchuk A, Ziai W, Anderson CS. 50.Intracerebral haemorrhage: current approaches to acute management. Lancet. 2018;392:1257–68. 10.1016/s0140-6736(18)31878-6. 30319113 10.1016/S0140-6736(18)31878-6

[56] Profaci CP, Munji RN, Pulido RS, Daneman R. The blood-brain barrier in health and disease: Important unanswered questions. J Exp Med. 2020. 10.1084/jem.20190062. 32211826 10.1084/jem.20190062 PMC7144528

[57] Wu X, Luo J, Liu H, Cui W, Guo K, Zhao L, et al. Correction to: recombinant adiponectin peptide ameliorates brain injury following intracerebral hemorrhage by suppressing astrocyte-derived inflammation via the inhibition of Drp1-mediated mitochondrial fission. Transl Stroke Res. 2023;14:1004. 10.1007/s12975-022-01085-6. 36169872 10.1007/s12975-022-01085-6

[58] Ronaldson PT, Davis TP. Regulation of blood-brain barrier integrity by microglia in health and disease: a therapeutic opportunity. J Cereb Blood Flow Metab. 2020;40:S6-s24. 10.1177/0271678x20951995. 32928017 10.1177/0271678X20951995 PMC7687032

[59] Lu Z, Wang Z, Yu L, Ding Y, Xu Y, Xu N, et al. 54.GCN2 reduces inflammation by p-eIF2α/ATF4 pathway after intracerebral hemorrhage in mice. Exp Neurol. 2019;313:16–25. 10.1016/j.expneurol.2018.12.004. 30529503 10.1016/j.expneurol.2018.12.004

[60] Tao J, Li J, Fan X, Jiang C, Wang Y, Qin M, et al. Unraveling the protein post-translational modification landscape: neuroinflammation and neuronal death after stroke. Ageing Res Rev. 2024;101:102489. 10.1016/j.arr.2024.102489. 39277050 10.1016/j.arr.2024.102489

[61] Zhang Z, Song Y, Li F, Xu Z, Huang Q. Inhibiting nuclear factor-κB at different stages after intracerebral hemorrhage can influence the hemorrhage-induced brain injury in experimental models in vivo. Brain Res Bull. 2020;155:159–65. 10.1016/j.brainresbull.2019.12.010. 31857135 10.1016/j.brainresbull.2019.12.010

[62] Tschoe C, Bushnell CD, Duncan PW, Alexander-Miller MA, Wolfe SQ. Neuroinflammation after intracerebral hemorrhage and potential therapeutic targets. J Stroke. 2020;22:29–46. 10.5853/jos.2019.02236. 32027790 10.5853/jos.2019.02236 PMC7005353

[63] Lan X, Han X, Li Q, Yang QW, Wang J. Modulators of microglial activation and polarization after intracerebral haemorrhage. Nat Rev Neurol. 2017;13:420–33. 10.1038/nrneurol.2017.69. 28524175 10.1038/nrneurol.2017.69 PMC5575938

[64] Gu L, Chen H, Geng R, Liang T, Chen Y, Wang Z, et al. Endothelial pyroptosis-driven microglial activation in choroid plexus mediates neuronal apoptosis in hemorrhagic stroke rats. Neurobiol Dis. 2024;201:106695. 10.1016/j.nbd.2024.106695. 39370051 10.1016/j.nbd.2024.106695

[65] Sun N, Wang H, Ma L, Lei P, Zhang Q. Ghrelin attenuates brain injury in septic mice via PI3K/Akt signaling activation. Brain Res Bull. 2016;124:278–85. 10.1016/j.brainresbull.2016.06.002. 27288247 10.1016/j.brainresbull.2016.06.002

[66] Guo M, Qiu MY, Zeng L, Nie YX, Tang YL, Luo Y, et al. Acidosis induces autophagic cell death through ASIC1-mediated Akt/mTOR signaling in HT22 neurons. Toxicology. 2025;511:154045. 10.1016/j.tox.2025.154045. 39756784 10.1016/j.tox.2025.154045

[67] Fang B, Zhao Q, Ling W, Zhang Y, Ou M. Hypoxia induces HT-22 neuronal cell death via Orai1/CDK5 pathway-mediated Tau hyperphosphorylation. Am J Transl Res. 2019;11:7591–603. 31934303 PMC6943478

