# Supplementary material for: Eerdun Wurile, a Mongolian medicine, alleviates neuronal damage and improves neurological dysfunction after cerebral hemorrhage by activating the PI3K/AKT signaling pathway
Source: Chin Med. 2026 Jul 7;21:181. doi: 10.1186/s13020-026-01459-0 (PMC13340406; doi:10.1186/s13020-026-01459-0)
Supplement: Supplementary file 1 — Additional file 1. [file 13020_2026_1459_MOESM1_ESM.docx]

**1. *Reagents and instruments***

1.1 ***Reagents***

**Table S1. Reagents and reference standards information sheet**


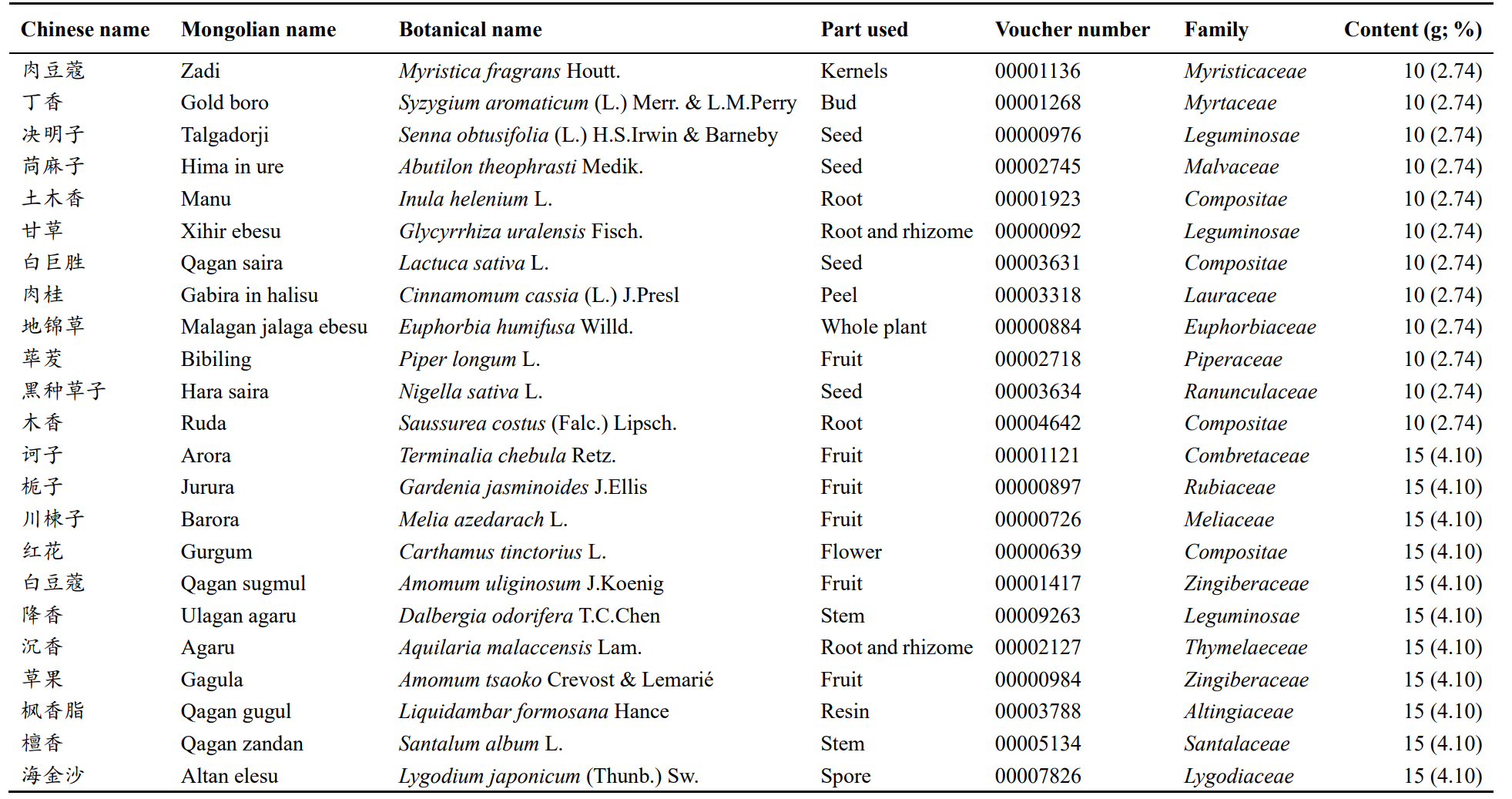


Note: Eerdun Wurile also contains 22.02% non-plant-based substances.

***2. Methods***

**2.1 *Metabolites Extraction***

I. Sample Extraction

Mix the sample thoroughly, then centrifuge at 12,000 r/min for 10 min. Accurately transfer approximately 100 μL of the supernatant into a 2 mL centrifuge tube, add 0.9 mL of methanol for dilution, and mix well. Filter the solution through a 0.22 μm membrane filter before HPLC analysis.

II. Instrument Conditions

Detector: VWD

Chromatographic column: Agilent C18, 250 × 4.6 mm, 5 μm

Column temperature: 30°C

Wavelength: 205 nm

Flow rate: 1 mL/min

Injection volume: 10 μL

Mobile phase: Methanol

III. Preparation of Standard Solutions

Accurately weigh a certain amount of each standard substance into a 5 mL brown volumetric flask. Add an appropriate amount of methanol, dissolve by ultrasonication, then dilute to the mark with methanol. Shake well to obtain a single-standard stock solution at a concentration of 2 mg/mL. Store at −20°C.

Precisely transfer a certain volume of each single-standard stock solution into a 5 mL brown volumetric flask, dilute to the mark with methanol, and shake well to obtain a mixed standard intermediate solution at various concentrations (see standard curve for details). Store at −20°C.

The standard working curve is prepared using methanol as the solvent.


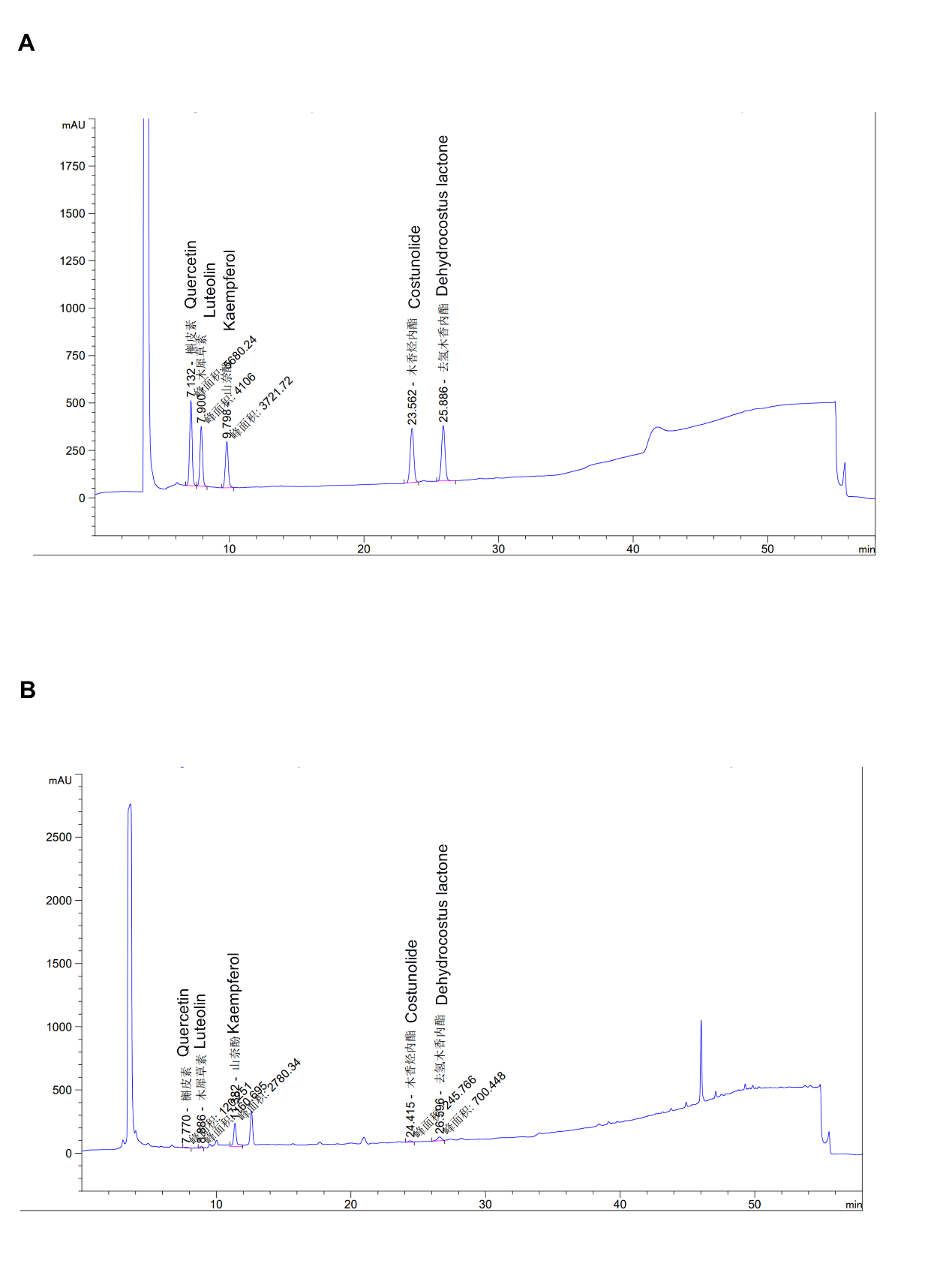


Figure S1: Representative HPLC chromatogram of EW, (A) reference standard; (B) sample.


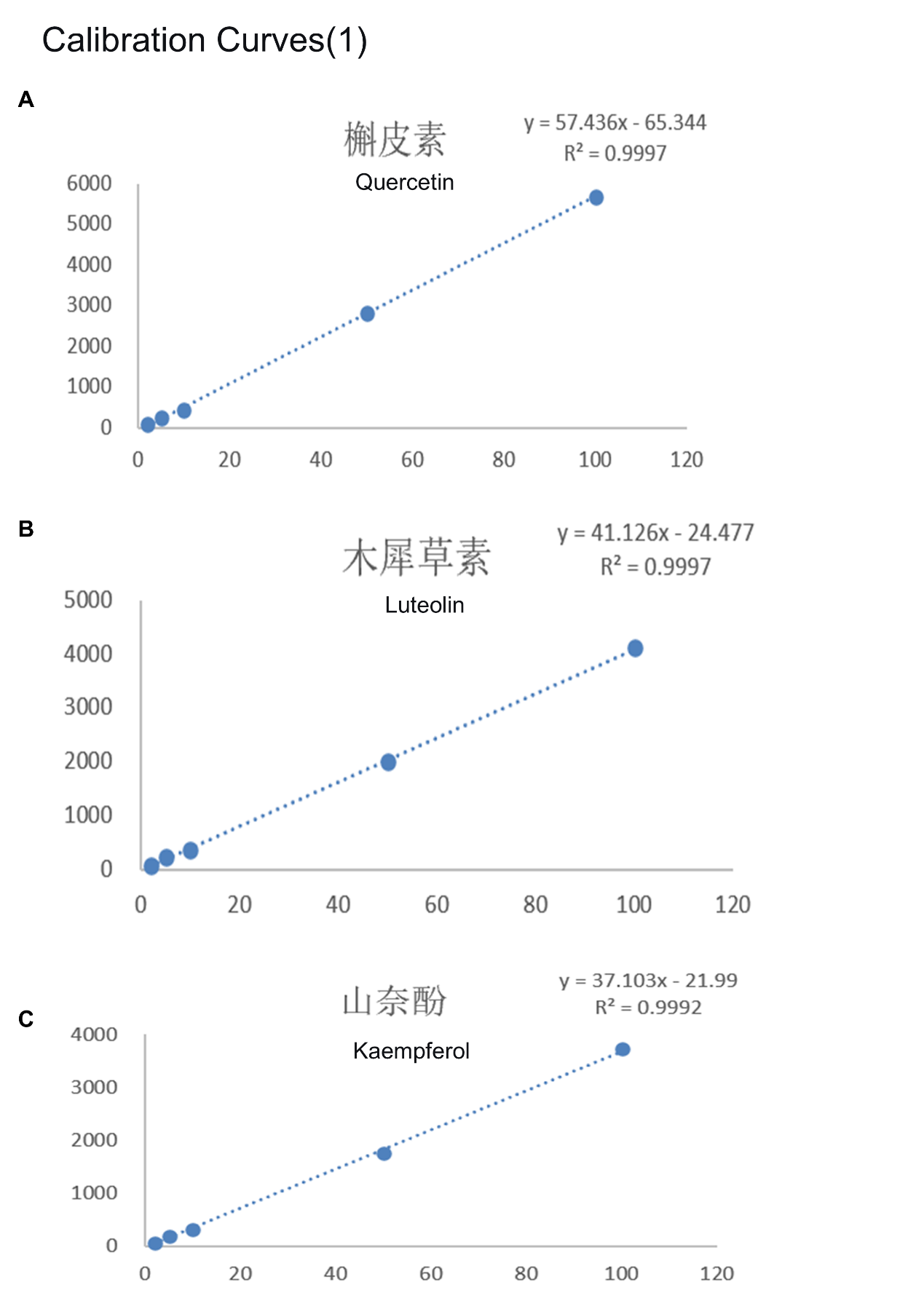


Figure S2: Calibration curves (1) of the detected components.


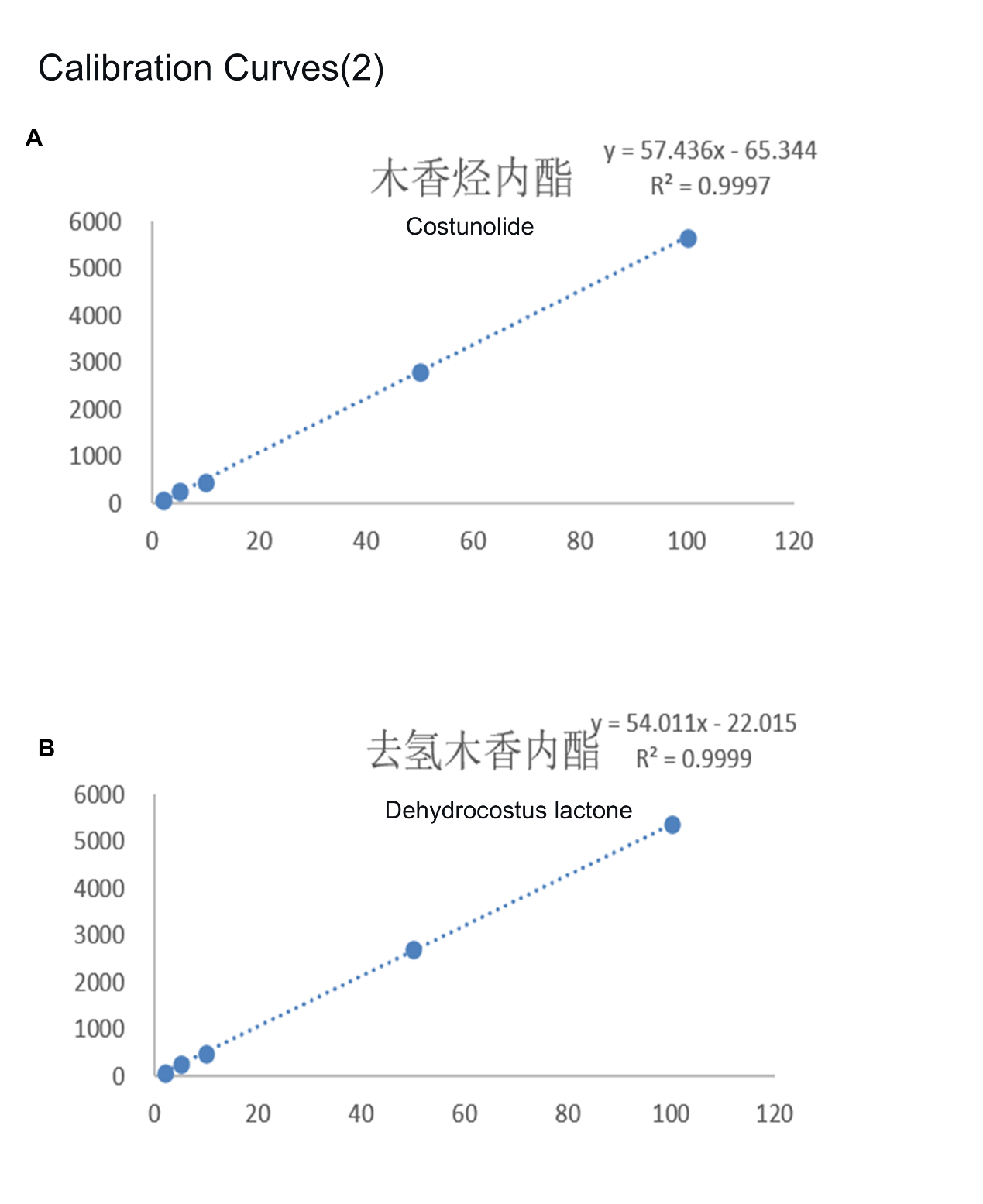


Figure S3: Calibration curves (2) of the detected components.


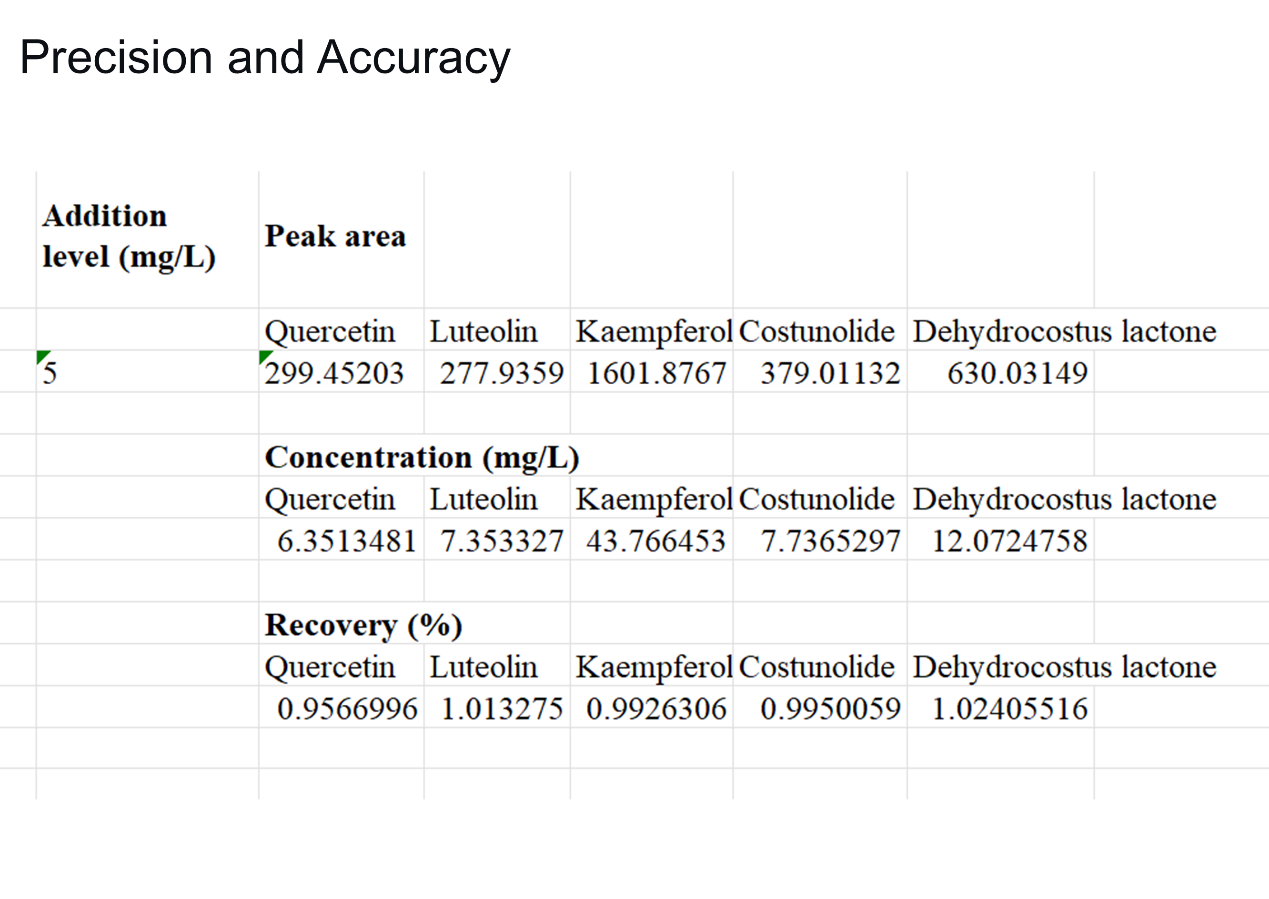


Figure S4: The quantitative results show the concentrations and recoveries of the samples.


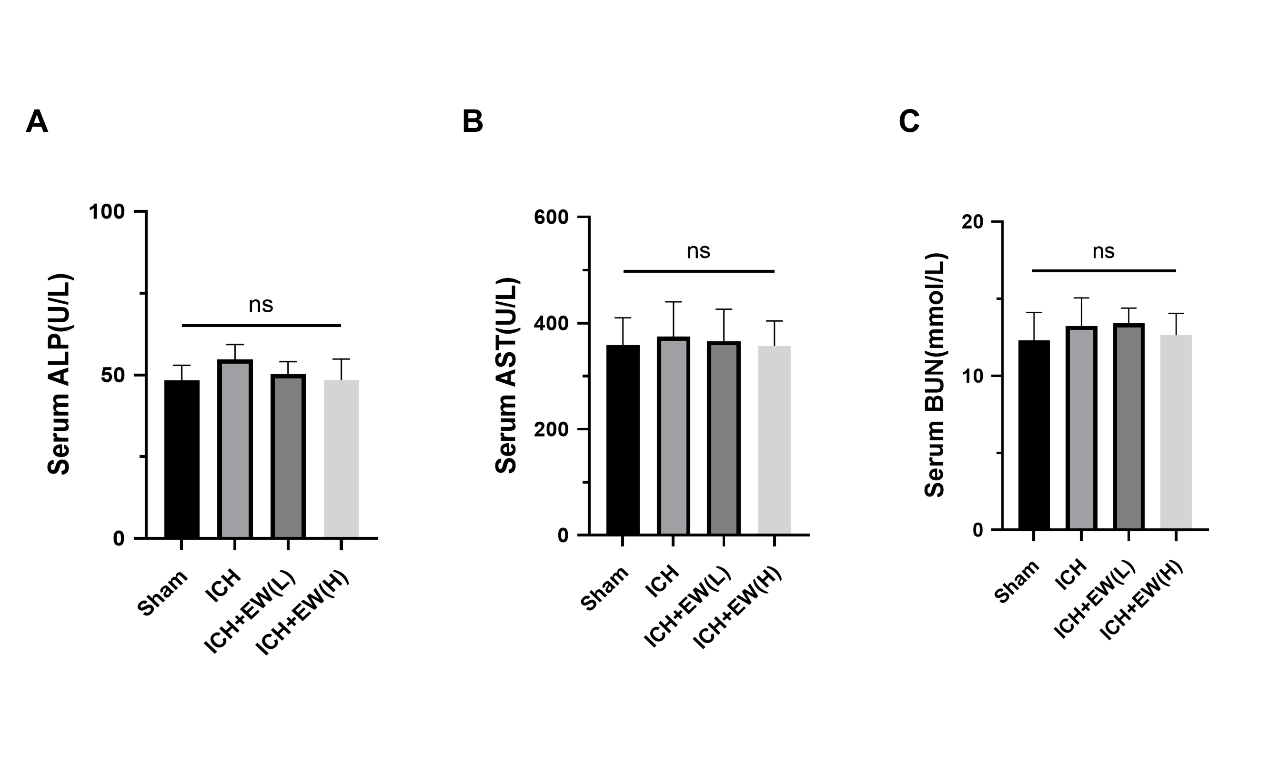


Figure S5: Safety assessment data.


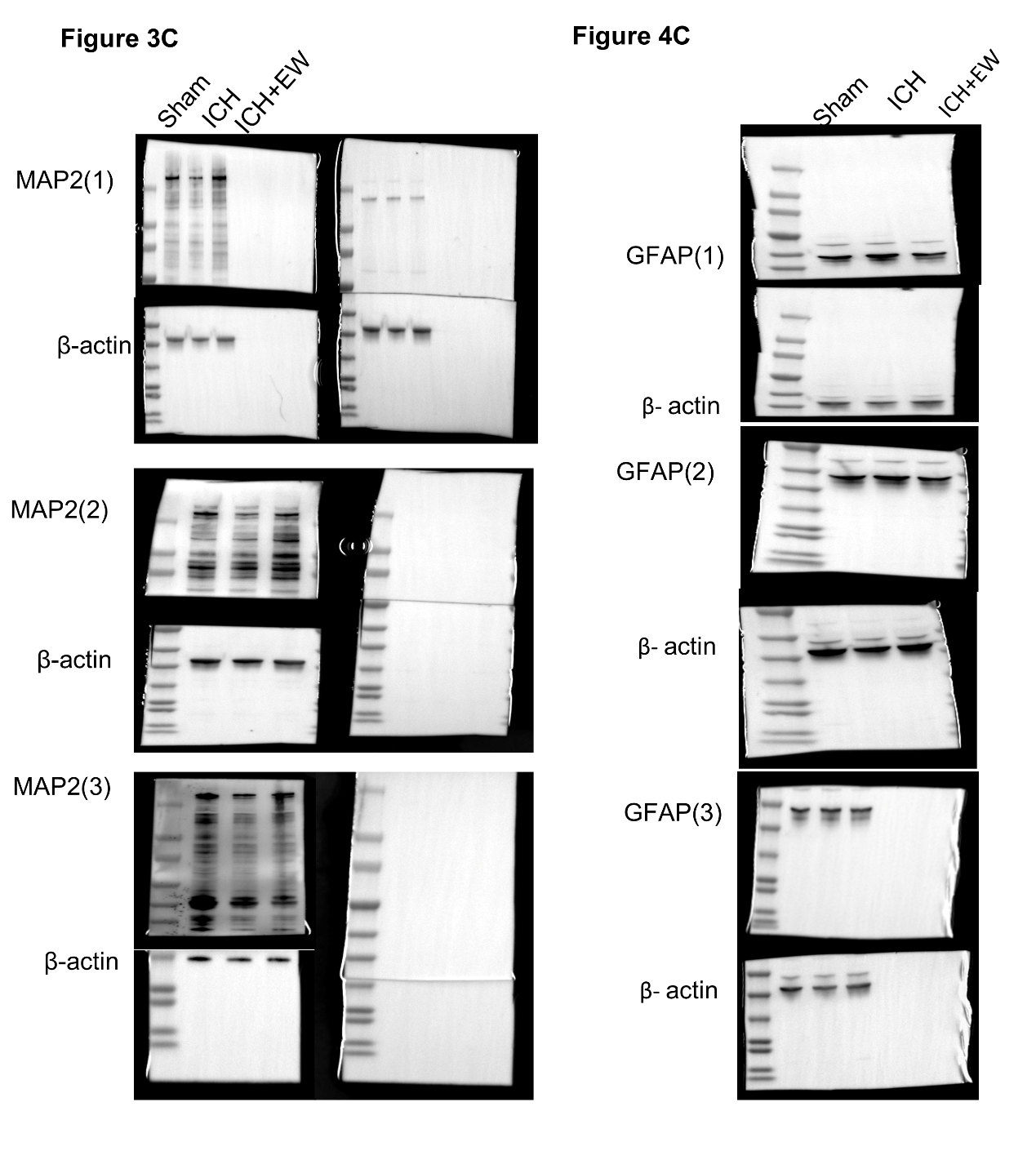


Figure S6


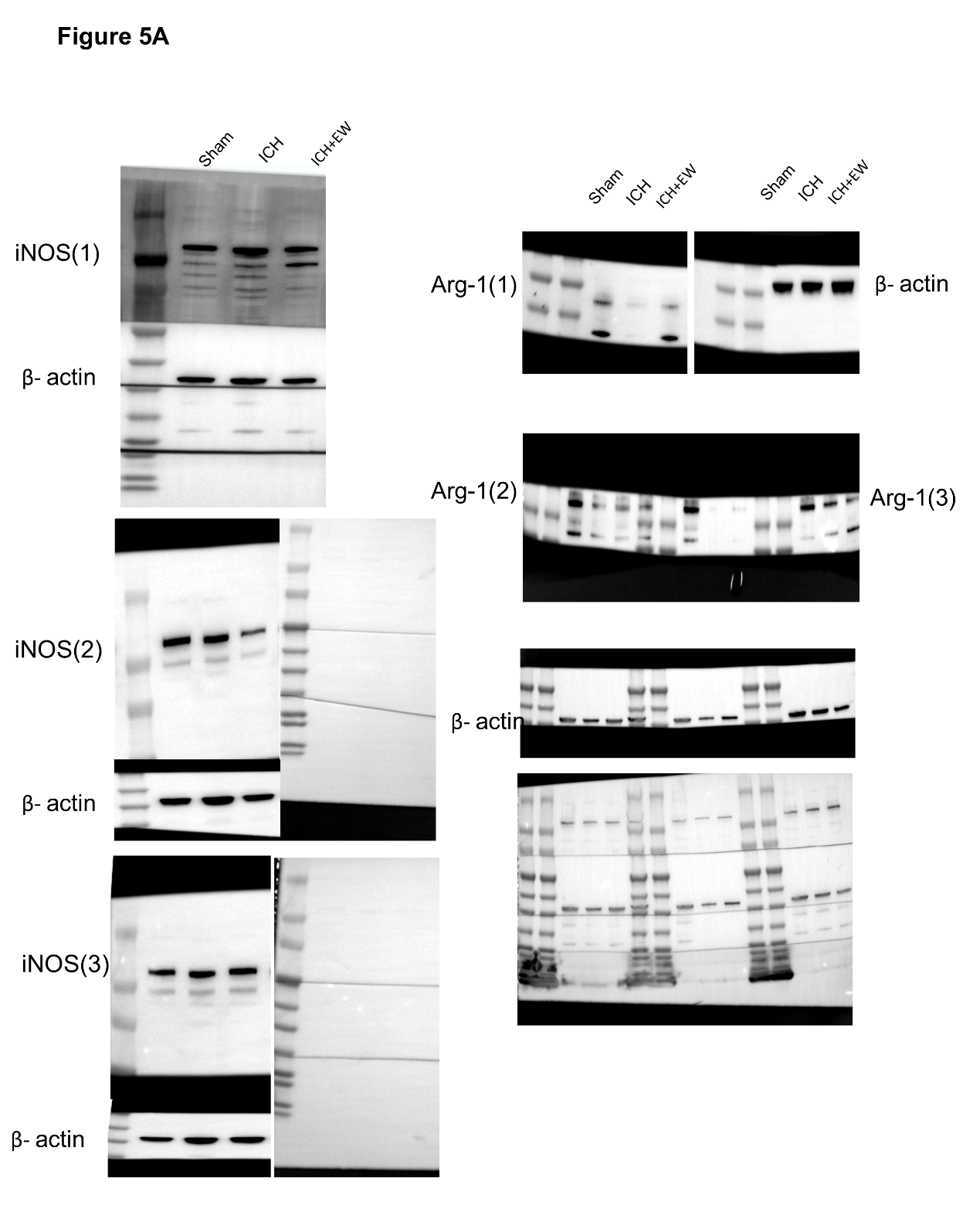


Figure S7


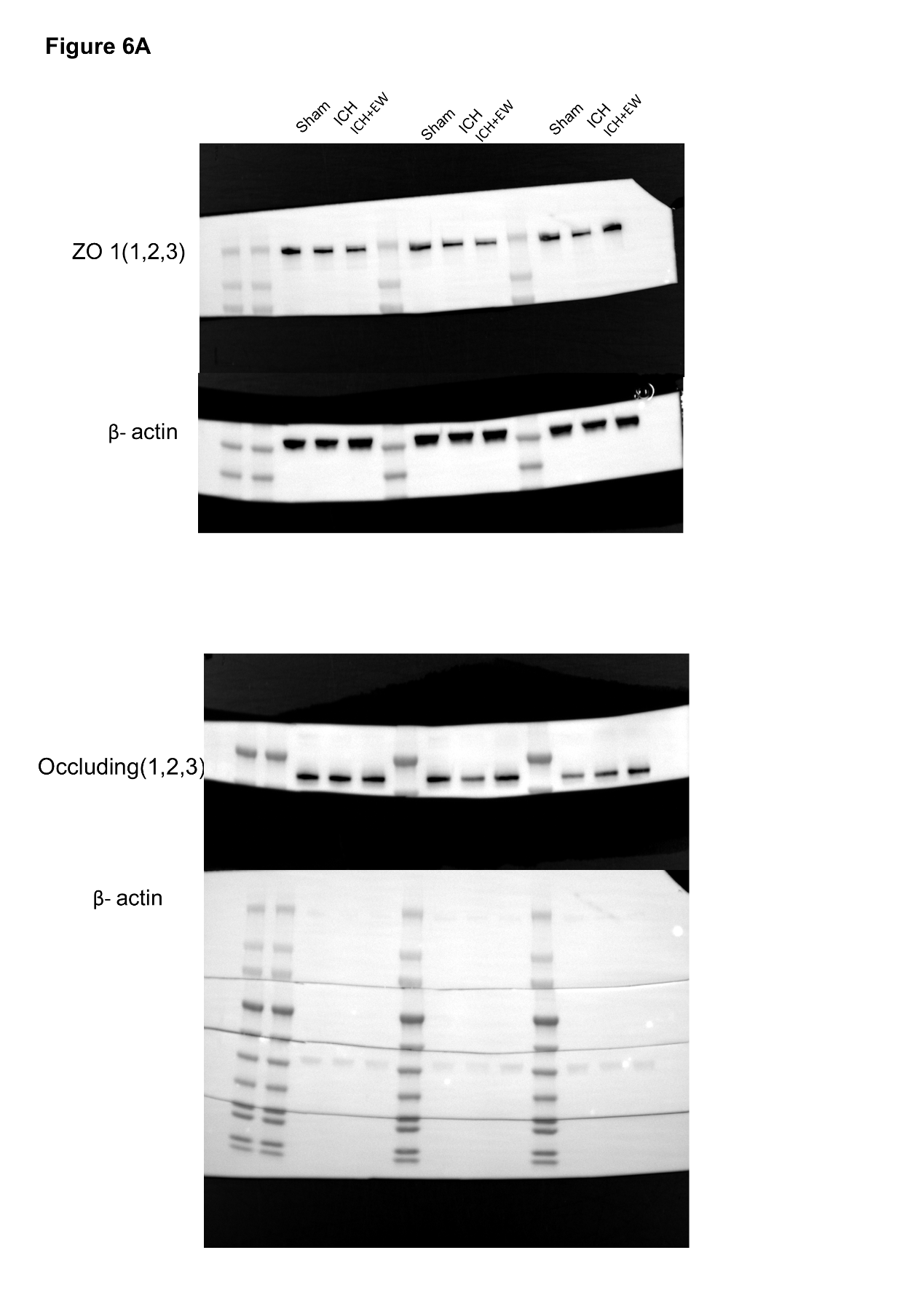


Figure S8


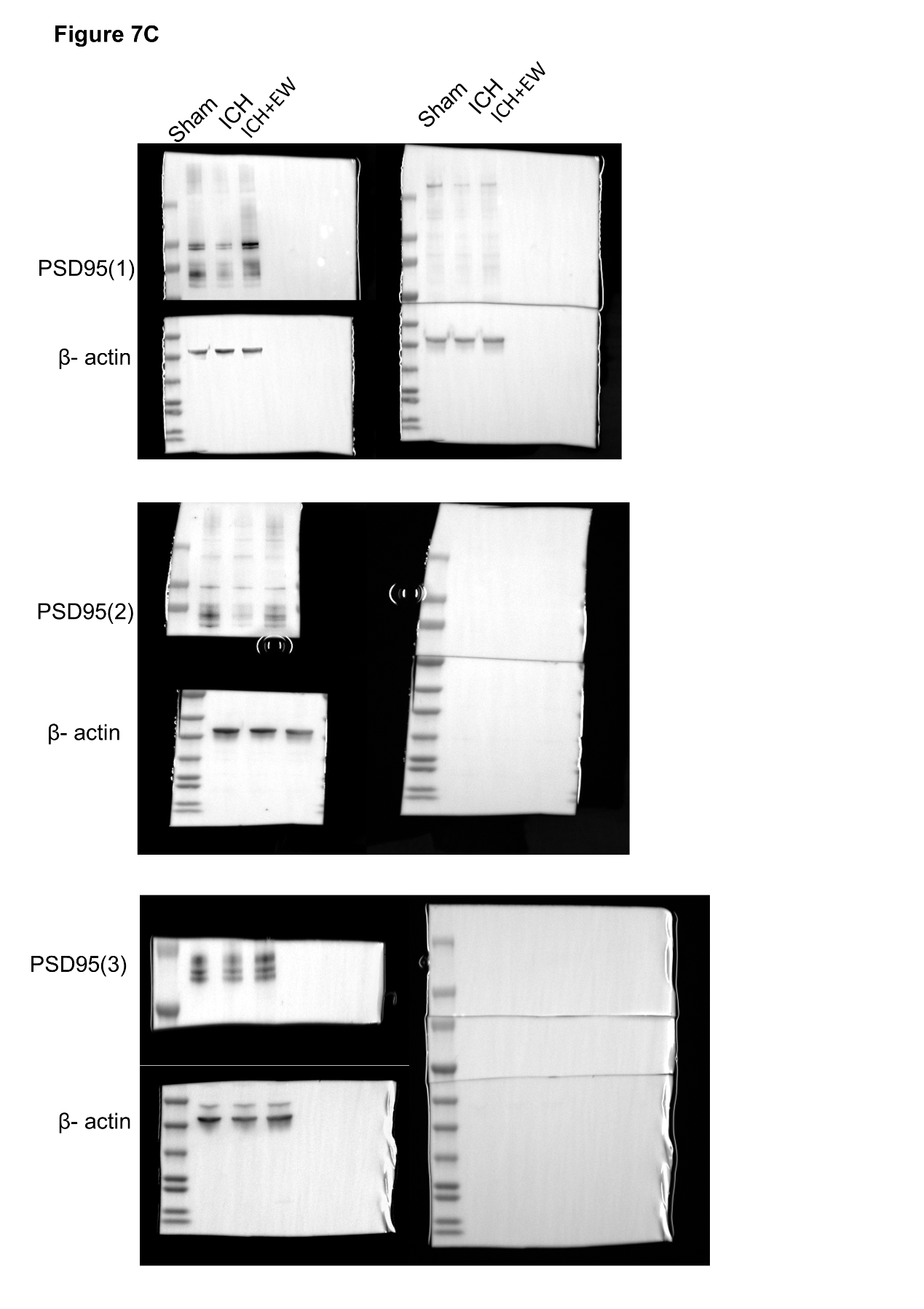


Figure S9


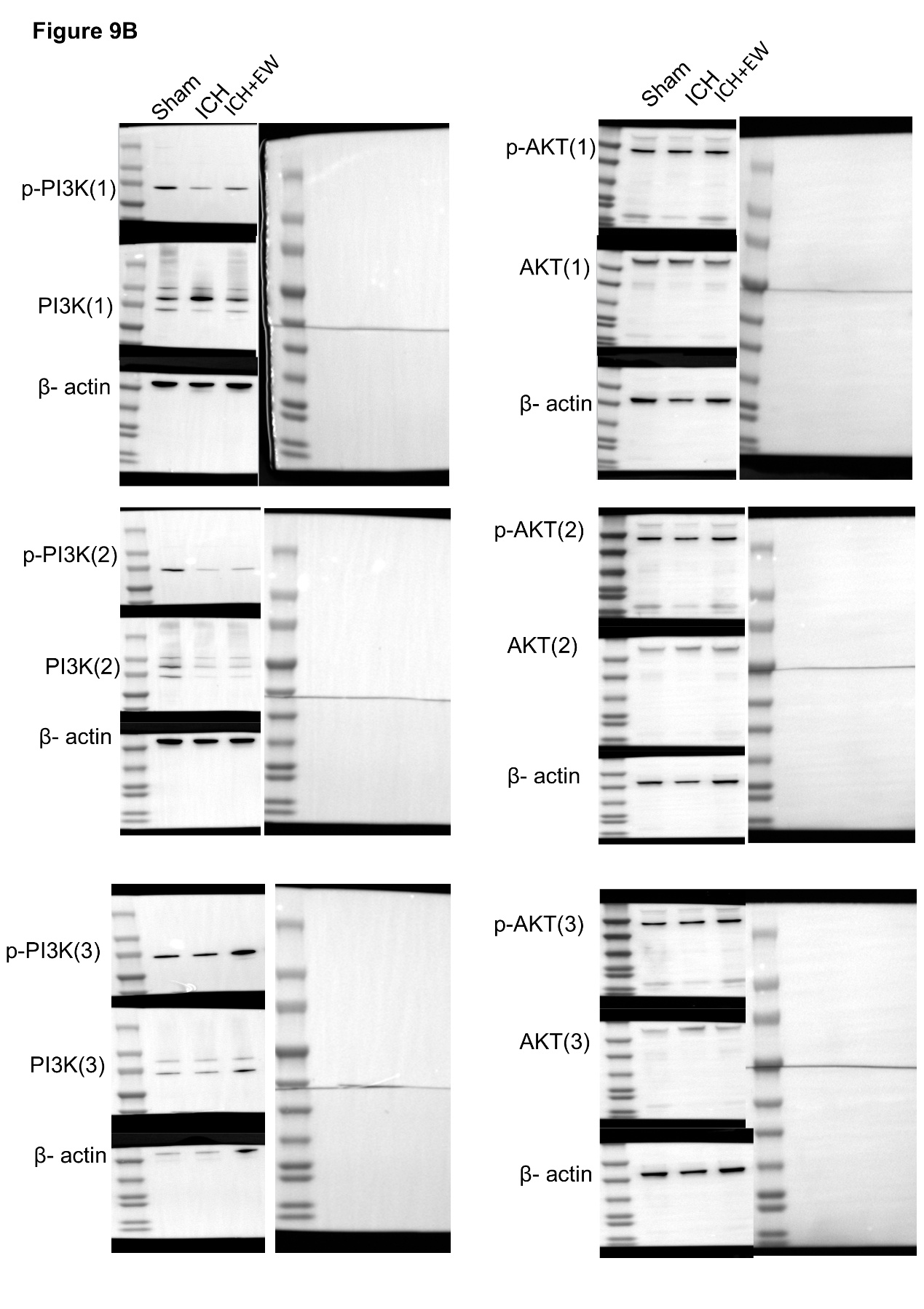


Figure S10


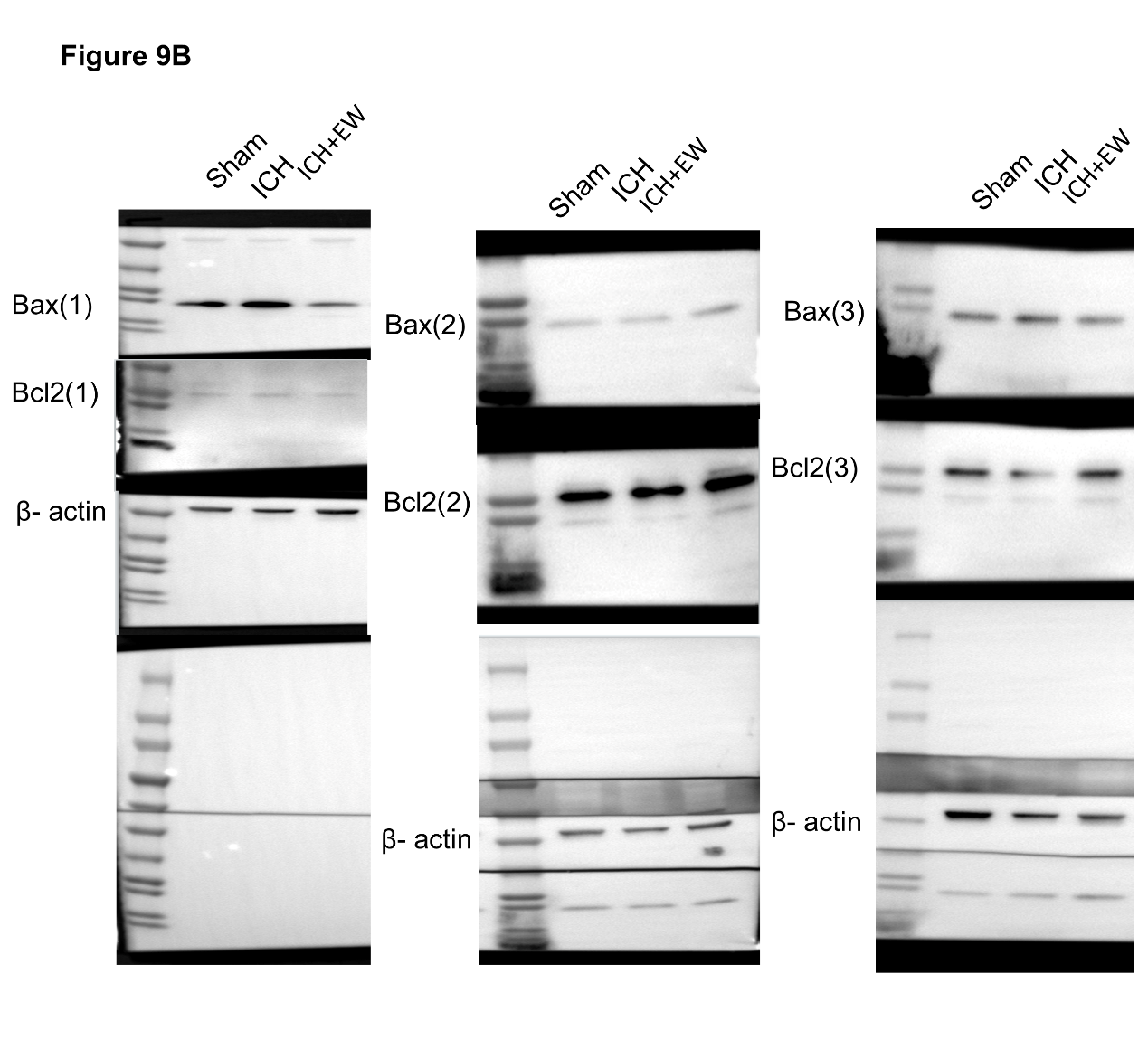


Figure S11


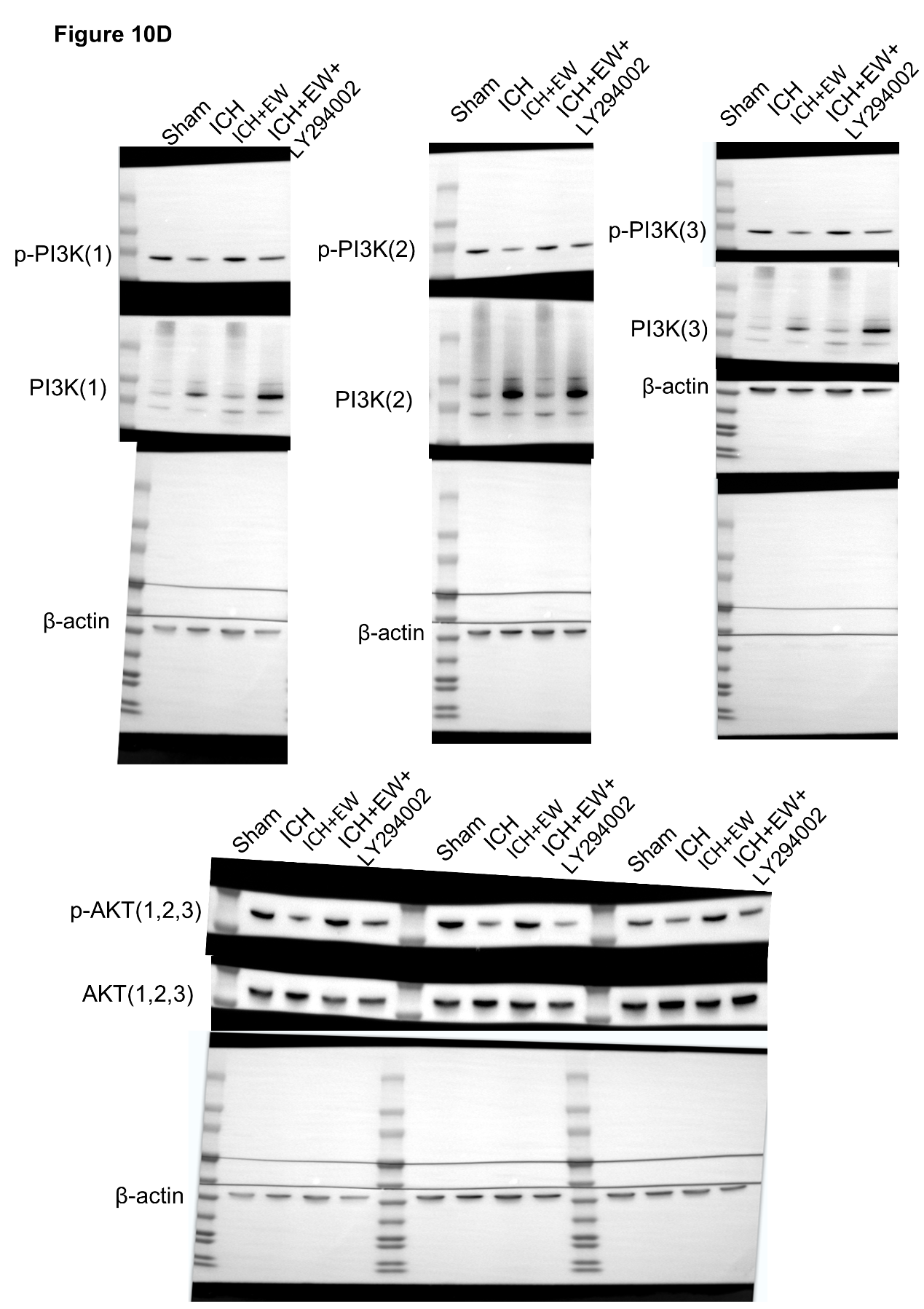


Figure S12


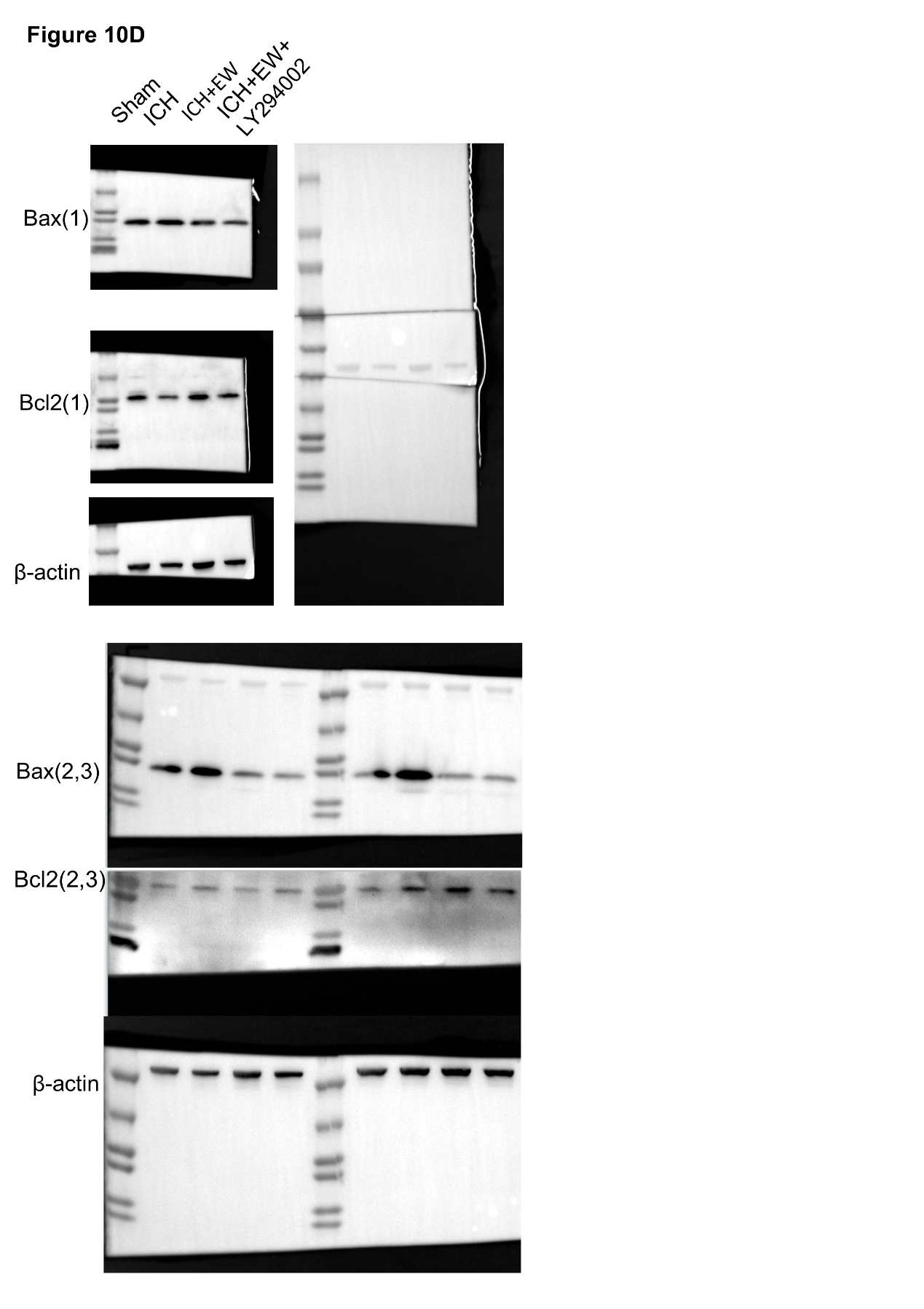


Figure S13


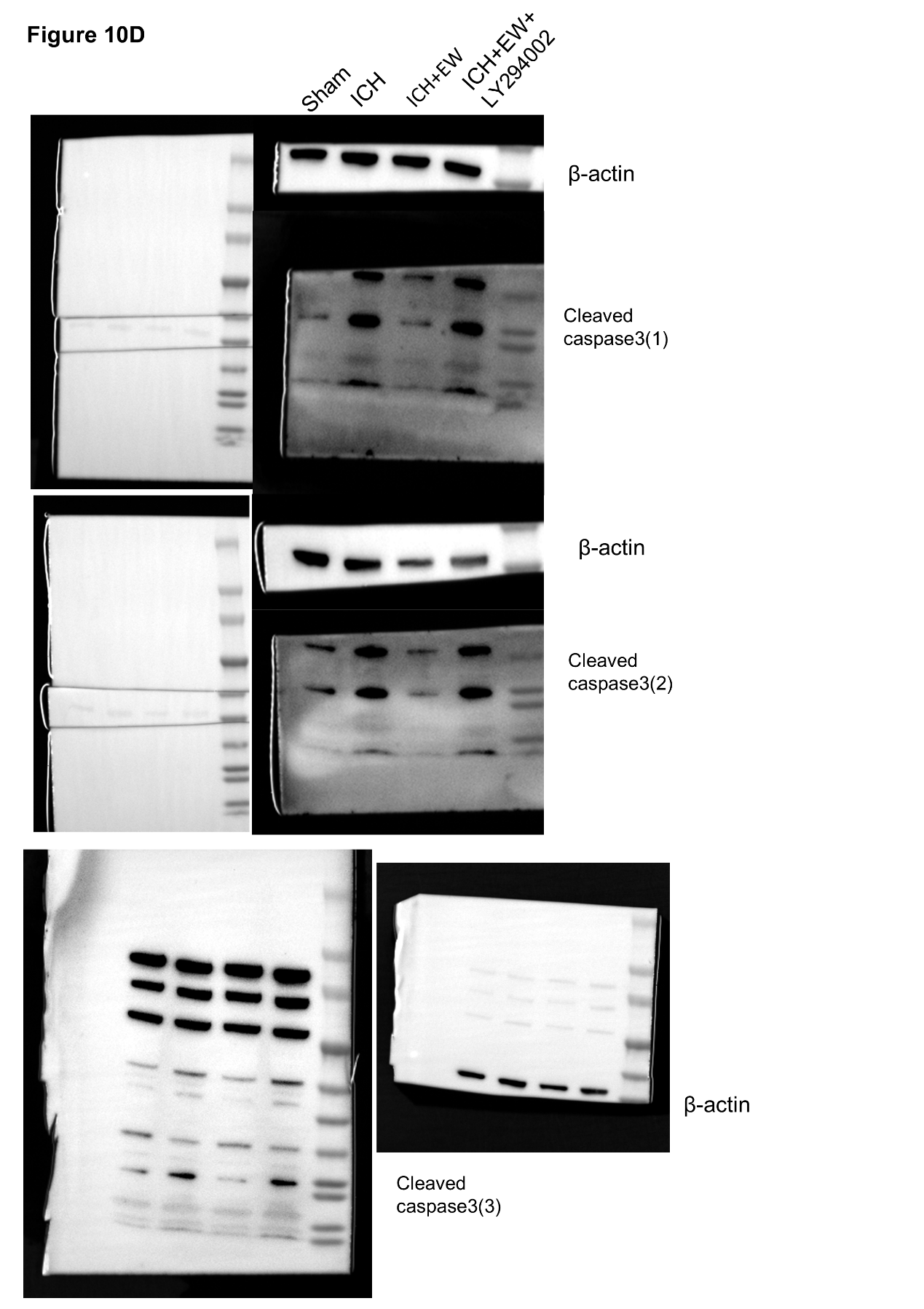


Figure S14
